# Retrospective evaluation of whole exome and genome mutation calls in 746 cancer samples

**DOI:** 10.1038/s41467-020-18151-y

**Published:** 2020-09-21

**Authors:** Matthew H. Bailey, William U. Meyerson, Lewis Jonathan Dursi, Liang-Bo Wang, Guanlan Dong, Wen-Wei Liang, Amila Weerasinghe, Shantao Li, Sean Kelso, MC3 Working Group, Rehan Akbani, Pavana Anur, Matthew H. Bailey, Alex Buchanan, Kami Chiotti, Kyle Covington, Allison Creason, Li Ding, Kyle Ellrott, Yu Fan, Steven Foltz, Gad Getz, Walker Hale, David Haussler, Julian M. Hess, Carolyn M. Hutter, Cyriac Kandoth, Katayoon Kasaian, Melpomeni Kasapi, Dave Larson, Ignaty Leshchiner, John Letaw, Singer Ma, Michael D. McLellan, Yifei Men, Gordon B. Mills, Beifang Niu, Myron Peto, Amie Radenbaugh, Sheila M. Reynolds, Gordon Saksena, Heidi Sofia, Chip Stewart, Adam J. Struck, Joshua M. Stuart, Wenyi Wang, John N. Weinstein, David A. Wheeler, Christopher K. Wong, Liu Xi, Kai Ye, PCAWG novel somatic mutation calling methods working group, Matthew H. Bailey, Beifang Niu, Matthias Bieg, Paul C. Boutros, Ivo Buchhalter, Adam P. Butler, Ken Chen, Zechen Chong, Li Ding, Oliver Drechsel, Lewis Jonathan Dursi, Roland Eils, Kyle Ellrott, Shadrielle M. G. Espiritu, Yu Fan, Robert S. Fulton, Shengjie Gao, Josep L. l. Gelpi, Mark B. Gerstein, Gad Getz, Santiago Gonzalez, Ivo G. Gut, Faraz Hach, Michael C. Heinold, Julian M. Hess, Jonathan Hinton, Taobo Hu, Vincent Huang, Yi Huang, Barbara Hutter, David R. Jones, Jongsun Jung, Natalie Jäger, Hyung-Lae Kim, Kortine Kleinheinz, Sushant Kumar, Yogesh Kumar, Christopher M. Lalansingh, Ignaty Leshchiner, Ivica Letunic, Dimitri Livitz, Eric Z. Ma, Yosef E. Maruvka, R. Jay Mashl, Michael D. McLellan, Andrew Menzies, Ana Milovanovic, Morten Muhlig Nielsen, Stephan Ossowski, Nagarajan Paramasivam, Jakob Skou Pedersen, Marc D. Perry, Montserrat Puiggròs, Keiran M. Raine, Esther Rheinbay, Romina Royo, S. Cenk Sahinalp, Gordon Saksena, Iman Sarrafi, Matthias Schlesner, Jared T. Simpson, Lucy Stebbings, Chip Stewart, Miranda D. Stobbe, Jon W. Teague, Grace Tiao, David Torrents, Jeremiah A. Wala, Jiayin Wang, Wenyi Wang, Sebastian M. Waszak, Joachim Weischenfeldt, Michael C. Wendl, Johannes Werner, Zhenggang Wu, Hong Xue, Sergei Yakneen, Takafumi N. Yamaguchi, Kai Ye, Venkata D. Yellapantula, Christina K. Yung, Junjun Zhang, Gordon Saksena, Kyle Ellrott, Michael C. Wendl, David A. Wheeler, Gad Getz, Jared T. Simpson, Mark B. Gerstein, Li Ding, PCAWG Consortium, Lauri A. Aaltonen, Federico Abascal, Adam Abeshouse, Hiroyuki Aburatani, David J. Adams, Nishant Agrawal, Keun Soo Ahn, Sung-Min Ahn, Hiroshi Aikata, Rehan Akbani, Kadir C. Akdemir, Hikmat Al-Ahmadie, Sultan T. Al-Sedairy, Fatima Al-Shahrour, Malik Alawi, Monique Albert, Kenneth Aldape, Ludmil B. Alexandrov, Adrian Ally, Kathryn Alsop, Eva G. Alvarez, Fernanda Amary, Samirkumar B. Amin, Brice Aminou, Ole Ammerpohl, Matthew J. Anderson, Yeng Ang, Davide Antonello, Pavana Anur, Samuel Aparicio, Elizabeth L. Appelbaum, Yasuhito Arai, Axel Aretz, Koji Arihiro, Shun-ichi Ariizumi, Joshua Armenia, Laurent Arnould, Sylvia Asa, Yassen Assenov, Gurnit Atwal, Sietse Aukema, J. Todd Auman, Miriam R. Aure, Philip Awadalla, Marta Aymerich, Gary D. Bader, Adrian Baez-Ortega, Matthew H. Bailey, Peter J. Bailey, Miruna Balasundaram, Saianand Balu, Pratiti Bandopadhayay, Rosamonde E. Banks, Stefano Barbi, Andrew P. Barbour, Jonathan Barenboim, Jill Barnholtz-Sloan, Hugh Barr, Elisabet Barrera, John Bartlett, Javier Bartolome, Claudio Bassi, Oliver F. Bathe, Daniel Baumhoer, Prashant Bavi, Stephen B. Baylin, Wojciech Bazant, Duncan Beardsmore, Timothy A. Beck, Sam Behjati, Andreas Behren, Beifang Niu, Cindy Bell, Sergi Beltran, Christopher Benz, Andrew Berchuck, Anke K. Bergmann, Erik N. Bergstrom, Benjamin P. Berman, Daniel M. Berney, Stephan H. Bernhart, Rameen Beroukhim, Mario Berrios, Samantha Bersani, Johanna Bertl, Miguel Betancourt, Vinayak Bhandari, Shriram G. Bhosle, Andrew V. Biankin, Matthias Bieg, Darell Bigner, Hans Binder, Ewan Birney, Michael Birrer, Nidhan K. Biswas, Bodil Bjerkehagen, Tom Bodenheimer, Lori Boice, Giada Bonizzato, Johann S. De Bono, Arnoud Boot, Moiz S. Bootwalla, Ake Borg, Arndt Borkhardt, Keith A. Boroevich, Ivan Borozan, Christoph Borst, Marcus Bosenberg, Mattia Bosio, Jacqueline Boultwood, Guillaume Bourque, Paul C. Boutros, G. Steven Bova, David T. Bowen, Reanne Bowlby, David D. L. Bowtell, Sandrine Boyault, Rich Boyce, Jeffrey Boyd, Alvis Brazma, Paul Brennan, Daniel S. Brewer, Arie B. Brinkman, Robert G. Bristow, Russell R. Broaddus, Jane E. Brock, Malcolm Brock, Annegien Broeks, Angela N. Brooks, Denise Brooks, Benedikt Brors, Søren Brunak, Timothy J. C. Bruxner, Alicia L. Bruzos, Alex Buchanan, Ivo Buchhalter, Christiane Buchholz, Susan Bullman, Hazel Burke, Birgit Burkhardt, Kathleen H. Burns, John Busanovich, Carlos D. Bustamante, Adam P. Butler, Atul J. Butte, Niall J. Byrne, Anne-Lise Børresen-Dale, Samantha J. Caesar-Johnson, Andy Cafferkey, Declan Cahill, Claudia Calabrese, Carlos Caldas, Fabien Calvo, Niedzica Camacho, Peter J. Campbell, Elias Campo, Cinzia Cantù, Shaolong Cao, Thomas E. Carey, Joana Carlevaro-Fita, Rebecca Carlsen, Ivana Cataldo, Mario Cazzola, Jonathan Cebon, Robert Cerfolio, Dianne E. Chadwick, Dimple Chakravarty, Don Chalmers, Calvin Wing Yiu Chan, Kin Chan, Michelle Chan-Seng-Yue, Vishal S. Chandan, David K. Chang, Stephen J. Chanock, Lorraine A. Chantrill, Aurélien Chateigner, Nilanjan Chatterjee, Kazuaki Chayama, Hsiao-Wei Chen, Jieming Chen, Ken Chen, Yiwen Chen, Zhaohong Chen, Andrew D. Cherniack, Jeremy Chien, Yoke-Eng Chiew, Suet-Feung Chin, Juok Cho, Sunghoon Cho, Jung Kyoon Choi, Wan Choi, Christine Chomienne, Zechen Chong, Su Pin Choo, Angela Chou, Angelika N. Christ, Elizabeth L. Christie, Eric Chuah, Carrie Cibulskis, Kristian Cibulskis, Sara Cingarlini, Peter Clapham, Alexander Claviez, Sean Cleary, Nicole Cloonan, Marek Cmero, Colin C. Collins, Ashton A. Connor, Susanna L. Cooke, Colin S. Cooper, Leslie Cope, Vincenzo Corbo, Matthew G. Cordes, Stephen M. Cordner, Isidro Cortés-Ciriano, Kyle Covington, Prue A. Cowin, Brian Craft, David Craft, Chad J. Creighton, Yupeng Cun, Erin Curley, Ioana Cutcutache, Karolina Czajka, Bogdan Czerniak, Rebecca A. Dagg, Ludmila Danilova, Maria Vittoria Davi, Natalie R. Davidson, Helen Davies, Ian J. Davis, Brandi N. Davis-Dusenbery, Kevin J. Dawson, Francisco M. De La Vega, Ricardo De Paoli-Iseppi, Timothy Defreitas, Angelo P. Dei Tos, Olivier Delaneau, John A. Demchok, Jonas Demeulemeester, German M. Demidov, Deniz Demircioğlu, Nening M. Dennis, Robert E. Denroche, Stefan C. Dentro, Nikita Desai, Vikram Deshpande, Amit G. Deshwar, Christine Desmedt, Jordi Deu-Pons, Noreen Dhalla, Neesha C. Dhani, Priyanka Dhingra, Rajiv Dhir, Anthony DiBiase, Klev Diamanti, Li Ding, Shuai Ding, Huy Q. Dinh, Luc Dirix, HarshaVardhan Doddapaneni, Nilgun Donmez, Michelle T. Dow, Ronny Drapkin, Oliver Drechsel, Ruben M. Drews, Serge Serge, Tim Dudderidge, Ana Dueso-Barroso, Andrew J. Dunford, Michael Dunn, Lewis Jonathan Dursi, Fraser R. Duthie, Ken Dutton-Regester, Jenna Eagles, Douglas F. Easton, Stuart Edmonds, Paul A. Edwards, Sandra E. Edwards, Rosalind A. Eeles, Anna Ehinger, Juergen Eils, Roland Eils, Adel El-Naggar, Matthew Eldridge, Kyle Ellrott, Serap Erkek, Georgia Escaramis, Shadrielle M. G. Espiritu, Xavier Estivill, Dariush Etemadmoghadam, Jorunn E. Eyfjord, Bishoy M. Faltas, Daiming Fan, Yu Fan, William C. Faquin, Claudiu Farcas, Matteo Fassan, Aquila Fatima, Francesco Favero, Nodirjon Fayzullaev, Ina Felau, Sian Fereday, Martin L. Ferguson, Vincent Ferretti, Lars Feuerbach, Matthew A. Field, J. Lynn Fink, Gaetano Finocchiaro, Cyril Fisher, Matthew W. Fittall, Anna Fitzgerald, Rebecca C. Fitzgerald, Adrienne M. Flanagan, Neil E. Fleshner, Paul Flicek, John A. Foekens, Kwun M. Fong, Nuno A. Fonseca, Christopher S. Foster, Natalie S. Fox, Michael Fraser, Scott Frazer, Milana Frenkel-Morgenstern, William Friedman, Joan Frigola, Catrina C. Fronick, Akihiro Fujimoto, Masashi Fujita, Masashi Fukayama, Lucinda A. Fulton, Robert S. Fulton, Mayuko Furuta, P. Andrew Futreal, Anja Füllgrabe, Stacey B. Gabriel, Steven Gallinger, Carlo Gambacorti-Passerini, Jianjiong Gao, Shengjie Gao, Levi Garraway, Øystein Garred, Erik Garrison, Dale W. Garsed, Nils Gehlenborg, Josep L. l. Gelpi, Joshy George, Daniela S. Gerhard, Clarissa Gerhauser, Jeffrey E. Gershenwald, Mark B. Gerstein, Moritz Gerstung, Gad Getz, Mohammed Ghori, Ronald Ghossein, Nasra H. Giama, Richard A. Gibbs, Anthony J. Gill, Pelvender Gill, Dilip D. Giri, Dominik Glodzik, Vincent J. Gnanapragasam, Maria Elisabeth Goebler, Mary J. Goldman, Carmen Gomez, Santiago Gonzalez, Abel Gonzalez-Perez, Dmitry A. Gordenin, James Gossage, Kunihito Gotoh, Ramaswamy Govindan, Dorthe Grabau, Janet S. Graham, Robert C. Grant, Anthony R. Green, Eric Green, Liliana Greger, Nicola Grehan, Sonia Grimaldi, Sean M. Grimmond, Robert L. Grossman, Adam Grundhoff, Gunes Gundem, Qianyun Guo, Manaswi Gupta, Shailja Gupta, Ivo G. Gut, Marta Gut, Jonathan Göke, Gavin Ha, Andrea Haake, David Haan, Siegfried Haas, Kerstin Haase, James E. Haber, Nina Habermann, Faraz Hach, Syed Haider, Natsuko Hama, Freddie C. Hamdy, Anne Hamilton, Mark P. Hamilton, Leng Han, George B. Hanna, Martin Hansmann, Nicholas J. Haradhvala, Olivier Harismendy, Ivon Harliwong, Arif O. Harmanci, Eoghan Harrington, Takanori Hasegawa, David Haussler, Steve Hawkins, Shinya Hayami, Shuto Hayashi, D. Neil Hayes, Stephen J. Hayes, Nicholas K. Hayward, Steven Hazell, Yao He, Allison P. Heath, Simon C. Heath, David Hedley, Apurva M. Hegde, David I. Heiman, Michael C. Heinold, Zachary Heins, Lawrence E. Heisler, Eva Hellstrom-Lindberg, Mohamed Helmy, Seong Gu Heo, Austin J. Hepperla, José María Heredia-Genestar, Carl Herrmann, Peter Hersey, Julian M. Hess, Holmfridur Hilmarsdottir, Jonathan Hinton, Satoshi Hirano, Nobuyoshi Hiraoka, Katherine A. Hoadley, Asger Hobolth, Ermin Hodzic, Jessica I. Hoell, Steve Hoffmann, Oliver Hofmann, Andrea Holbrook, Aliaksei Z. Holik, Michael A. Hollingsworth, Oliver Holmes, Robert A. Holt, Chen Hong, Eun Pyo Hong, Jongwhi H. Hong, Gerrit K. Hooijer, Henrik Hornshøj, Fumie Hosoda, Yong Hou, Volker Hovestadt, William Howat, Alan P. Hoyle, Ralph H. Hruban, Jianhong Hu, Taobo Hu, Xing Hua, Kuan-lin Huang, Mei Huang, Mi Ni Huang, Vincent Huang, Yi Huang, Wolfgang Huber, Thomas J. Hudson, Michael Hummel, Jillian A. Hung, David Huntsman, Ted R. Hupp, Jason Huse, Matthew R. Huska, Barbara Hutter, Carolyn M. Hutter, Daniel Hübschmann, Christine A. Iacobuzio-Donahue, Charles David Imbusch, Marcin Imielinski, Seiya Imoto, William B. Isaacs, Keren Isaev, Shumpei Ishikawa, Murat Iskar, S. M. Ashiqul Islam, Michael Ittmann, Sinisa Ivkovic, Jose M. G. Izarzugaza, Jocelyne Jacquemier, Valerie Jakrot, Nigel B. Jamieson, Gun Ho Jang, Se Jin Jang, Joy C. Jayaseelan, Reyka Jayasinghe, Stuart R. Jefferys, Karine Jegalian, Jennifer L. Jennings, Seung-Hyup Jeon, Lara Jerman, Yuan Ji, Wei Jiao, Peter A. Johansson, Amber L. Johns, Jeremy Johns, Rory Johnson, Todd A. Johnson, Clemency Jolly, Yann Joly, Jon G. Jonasson, Corbin D. Jones, David R. Jones, David T. W. Jones, Nic Jones, Steven J. M. Jones, Jos Jonkers, Young Seok Ju, Hartmut Juhl, Jongsun Jung, Malene Juul, Randi Istrup Juul, Sissel Juul, Natalie Jäger, Rolf Kabbe, Andre Kahles, Abdullah Kahraman, Vera B. Kaiser, Hojabr Kakavand, Sangeetha Kalimuthu, Christof von Kalle, Koo Jeong Kang, Katalin Karaszi, Beth Karlan, Rosa Karlić, Dennis Karsch, Katayoon Kasaian, Karin S. Kassahn, Hitoshi Katai, Mamoru Kato, Hiroto Katoh, Yoshiiku Kawakami, Jonathan D. Kay, Stephen H. Kazakoff, Marat D. Kazanov, Maria Keays, Electron Kebebew, Richard F. Kefford, Manolis Kellis, James G. Kench, Catherine J. Kennedy, Jules N. A. Kerssemakers, David Khoo, Vincent Khoo, Narong Khuntikeo, Ekta Khurana, Helena Kilpinen, Hark Kyun Kim, Hyung-Lae Kim, Hyung-Yong Kim, Hyunghwan Kim, Jaegil Kim, Jihoon Kim, Jong K. Kim, Youngwook Kim, Tari A. King, Wolfram Klapper, Kortine Kleinheinz, Leszek J. Klimczak, Stian Knappskog, Michael Kneba, Bartha M. Knoppers, Youngil Koh, Jan Komorowski, Daisuke Komura, Mitsuhiro Komura, Gu Kong, Marcel Kool, Jan O. Korbel, Viktoriya Korchina, Andrey Korshunov, Michael Koscher, Roelof Koster, Zsofia Kote-Jarai, Antonios Koures, Milena Kovacevic, Barbara Kremeyer, Helene Kretzmer, Markus Kreuz, Savitri Krishnamurthy, Dieter Kube, Kiran Kumar, Pardeep Kumar, Sushant Kumar, Yogesh Kumar, Ritika Kundra, Kirsten Kübler, Ralf Küppers, Jesper Lagergren, Phillip H. Lai, Peter W. Laird, Sunil R. Lakhani, Christopher M. Lalansingh, Emilie Lalonde, Fabien C. Lamaze, Adam Lambert, Eric Lander, Pablo Landgraf, Luca Landoni, Anita Langerød, Andrés Lanzós, Denis Larsimont, Erik Larsson, Mark Lathrop, Loretta M. S. Lau, Chris Lawerenz, Rita T. Lawlor, Michael S. Lawrence, Alexander J. Lazar, Xuan Le, Darlene Lee, Donghoon Lee, Eunjung Alice Lee, Hee Jin Lee, Jake June-Koo Lee, Jeong-Yeon Lee, Juhee Lee, Ming Ta Michael Lee, Henry Lee-Six, Kjong-Van Lehmann, Hans Lehrach, Dido Lenze, Conrad R. Leonard, Daniel A. Leongamornlert, Ignaty Leshchiner, Louis Letourneau, Ivica Letunic, Douglas A. Levine, Lora Lewis, Tim Ley, Chang Li, Constance H. Li, Haiyan Irene Li, Jun Li, Lin Li, Shantao Li, Siliang Li, Xiaobo Li, Xiaotong Li, Xinyue Li, Yilong Li, Han Liang, Sheng-Ben Liang, Peter Lichter, Pei Lin, Ziao Lin, W. M. Linehan, Ole Christian Lingjærde, Dongbing Liu, Eric Minwei Liu, Fei-Fei Liu, Fenglin Liu, Jia Liu, Xingmin Liu, Julie Livingstone, Dimitri Livitz, Naomi Livni, Lucas Lochovsky, Markus Loeffler, Georgina V. Long, Armando Lopez-Guillermo, Shaoke Lou, David N. Louis, Laurence B. Lovat, Yiling Lu, Yong-Jie Lu, Youyong Lu, Claudio Luchini, Ilinca Lungu, Xuemei Luo, Hayley J. Luxton, Andy G. Lynch, Lisa Lype, Cristina López, Carlos López-Otín, Eric Z. Ma, Yussanne Ma, Gaetan MacGrogan, Shona MacRae, Geoff Macintyre, Tobias Madsen, Kazuhiro Maejima, Andrea Mafficini, Dennis T. Maglinte, Arindam Maitra, Partha P. Majumder, Luca Malcovati, Salem Malikic, Giuseppe Malleo, Graham J. Mann, Luisa Mantovani-Löffler, Kathleen Marchal, Giovanni Marchegiani, Elaine R. Mardis, Adam A. Margolin, Maximillian G. Marin, Florian Markowetz, Julia Markowski, Jeffrey Marks, Tomas Marques-Bonet, Marco A. Marra, Luke Marsden, John W. M. Martens, Sancha Martin, Jose I. Martin-Subero, Iñigo Martincorena, Alexander Martinez-Fundichely, Yosef E. Maruvka, R. Jay Mashl, Charlie E. Massie, Thomas J. Matthew, Lucy Matthews, Erik Mayer, Simon Mayes, Michael Mayo, Faridah Mbabaali, Karen McCune, Ultan McDermott, Patrick D. McGillivray, Michael D. McLellan, John D. McPherson, John R. McPherson, Treasa A. McPherson, Samuel R. Meier, Alice Meng, Shaowu Meng, Andrew Menzies, Neil D. Merrett, Sue Merson, Matthew Meyerson, William U. Meyerson, Piotr A. Mieczkowski, George L. Mihaiescu, Sanja Mijalkovic, Ana Mijalkovic Mijalkovic-Lazic, Tom Mikkelsen, Michele Milella, Linda Mileshkin, Christopher A. Miller, David K. Miller, Jessica K. Miller, Gordon B. Mills, Ana Milovanovic, Sarah Minner, Marco Miotto, Gisela Mir Arnau, Lisa Mirabello, Chris Mitchell, Thomas J. Mitchell, Satoru Miyano, Naoki Miyoshi, Shinichi Mizuno, Fruzsina Molnár-Gábor, Malcolm J. Moore, Richard A. Moore, Sandro Morganella, Quaid D. Morris, Carl Morrison, Lisle E. Mose, Catherine D. Moser, Ferran Muiños, Loris Mularoni, Andrew J. Mungall, Karen Mungall, Elizabeth A. Musgrove, Ville Mustonen, David Mutch, Francesc Muyas, Donna M. Muzny, Alfonso Muñoz, Jerome Myers, Ola Myklebost, Peter Möller, Genta Nagae, Adnan M. Nagrial, Hardeep K. Nahal-Bose, Hitoshi Nakagama, Hidewaki Nakagawa, Hiromi Nakamura, Toru Nakamura, Kaoru Nakano, Tannistha Nandi, Jyoti Nangalia, Mia Nastic, Arcadi Navarro, Fabio C. P. Navarro, David E. Neal, Gerd Nettekoven, Felicity Newell, Steven J. Newhouse, Yulia Newton, Alvin Wei Tian Ng, Anthony Ng, Jonathan Nicholson, David Nicol, Yongzhan Nie, G. Petur Nielsen, Morten Muhlig Nielsen, Serena Nik-Zainal, Michael S. Noble, Katia Nones, Paul A. Northcott, Faiyaz Notta, Brian D. O’Connor, Peter O’Donnell, Maria O’Donovan, Sarah O’Meara, Brian Patrick O’Neill, J. Robert O’Neill, David Ocana, Angelica Ochoa, Layla Oesper, Christopher Ogden, Hideki Ohdan, Kazuhiro Ohi, Lucila Ohno-Machado, Karin A. Oien, Akinyemi I. Ojesina, Hidenori Ojima, Takuji Okusaka, Larsson Omberg, Choon Kiat Ong, Stephan Ossowski, German Ott, B. F. Francis Ouellette, Christine P’ng, Marta Paczkowska, Salvatore Paiella, Chawalit Pairojkul, Marina Pajic, Qiang Pan-Hammarström, Elli Papaemmanuil, Irene Papatheodorou, Nagarajan Paramasivam, Ji Wan Park, Joong-Won Park, Keunchil Park, Kiejung Park, Peter J. Park, Joel S. Parker, Simon L. Parsons, Harvey Pass, Danielle Pasternack, Alessandro Pastore, Ann-Marie Patch, Iris Pauporté, Antonio Pea, John V. Pearson, Chandra Sekhar Pedamallu, Jakob Skou Pedersen, Paolo Pederzoli, Martin Peifer, Nathan A. Pennell, Charles M. Perou, Marc D. Perry, Gloria M. Petersen, Myron Peto, Nicholas Petrelli, Robert Petryszak, Stefan M. Pfister, Mark Phillips, Oriol Pich, Hilda A. Pickett, Todd D. Pihl, Nischalan Pillay, Sarah Pinder, Mark Pinese, Andreia V. Pinho, Esa Pitkänen, Xavier Pivot, Elena Piñeiro-Yáñez, Laura Planko, Christoph Plass, Paz Polak, Tirso Pons, Irinel Popescu, Olga Potapova, Aparna Prasad, Shaun R. Preston, Manuel Prinz, Antonia L. Pritchard, Stephenie D. Prokopec, Elena Provenzano, Xose S. Puente, Sonia Puig, Montserrat Puiggròs, Sergio Pulido-Tamayo, Gulietta M. Pupo, Colin A. Purdie, Michael C. Quinn, Raquel Rabionet, Janet S. Rader, Bernhard Radlwimmer, Petar Radovic, Benjamin Raeder, Keiran M. Raine, Manasa Ramakrishna, Kamna Ramakrishnan, Suresh Ramalingam, Benjamin J. Raphael, W. Kimryn Rathmell, Tobias Rausch, Guido Reifenberger, Jüri Reimand, Jorge Reis-Filho, Victor Reuter, Iker Reyes-Salazar, Matthew A. Reyna, Sheila M. Reynolds, Esther Rheinbay, Yasser Riazalhosseini, Andrea L. Richardson, Julia Richter, Matthew Ringel, Markus Ringnér, Yasushi Rino, Karsten Rippe, Jeffrey Roach, Lewis R. Roberts, Nicola D. Roberts, Steven A. Roberts, A. Gordon Robertson, Alan J. Robertson, Javier Bartolomé Rodriguez, Bernardo Rodriguez-Martin, F. Germán Rodríguez-González, Michael H. A. Roehrl, Marius Rohde, Hirofumi Rokutan, Gilles Romieu, Ilse Rooman, Tom Roques, Daniel Rosebrock, Mara Rosenberg, Philip C. Rosenstiel, Andreas Rosenwald, Edward W. Rowe, Romina Royo, Steven G. Rozen, Yulia Rubanova, Mark A. Rubin, Carlota Rubio-Perez, Vasilisa A. Rudneva, Borislav C. Rusev, Andrea Ruzzenente, Gunnar Rätsch, Radhakrishnan Sabarinathan, Veronica Y. Sabelnykova, Sara Sadeghi, S. Cenk Sahinalp, Natalie Saini, Mihoko Saito-Adachi, Gordon Saksena, Adriana Salcedo, Roberto Salgado, Leonidas Salichos, Richard Sallari, Charles Saller, Roberto Salvia, Michelle Sam, Jaswinder S. Samra, Francisco Sanchez-Vega, Chris Sander, Grant Sanders, Rajiv Sarin, Iman Sarrafi, Aya Sasaki-Oku, Torill Sauer, Guido Sauter, Robyn P. M. Saw, Maria Scardoni, Christopher J. Scarlett, Aldo Scarpa, Ghislaine Scelo, Dirk Schadendorf, Jacqueline E. Schein, Markus B. Schilhabel, Matthias Schlesner, Thorsten Schlomm, Heather K. Schmidt, Sarah-Jane Schramm, Stefan Schreiber, Nikolaus Schultz, Steven E. Schumacher, Roland F. Schwarz, Richard A. Scolyer, David Scott, Ralph Scully, Raja Seethala, Ayellet V. Segre, Iris Selander, Colin A. Semple, Yasin Senbabaoglu, Subhajit Sengupta, Elisabetta Sereni, Stefano Serra, Dennis C. Sgroi, Mark Shackleton, Nimish C. Shah, Sagedeh Shahabi, Catherine A. Shang, Ping Shang, Ofer Shapira, Troy Shelton, Ciyue Shen, Hui Shen, Rebecca Shepherd, Ruian Shi, Yan Shi, Yu-Jia Shiah, Tatsuhiro Shibata, Juliann Shih, Eigo Shimizu, Kiyo Shimizu, Seung Jun Shin, Yuichi Shiraishi, Tal Shmaya, Ilya Shmulevich, Solomon I. Shorser, Charles Short, Raunak Shrestha, Suyash S. Shringarpure, Craig Shriver, Shimin Shuai, Nikos Sidiropoulos, Reiner Siebert, Anieta M. Sieuwerts, Lina Sieverling, Sabina Signoretti, Katarzyna O. Sikora, Michele Simbolo, Ronald Simon, Janae V. Simons, Jared T. Simpson, Peter T. Simpson, Samuel Singer, Nasa Sinnott-Armstrong, Payal Sipahimalani, Tara J. Skelly, Marcel Smid, Jaclyn Smith, Karen Smith-McCune, Nicholas D. Socci, Heidi J. Sofia, Matthew G. Soloway, Lei Song, Anil K. Sood, Sharmila Sothi, Christos Sotiriou, Cameron M. Soulette, Paul N. Span, Paul T. Spellman, Nicola Sperandio, Andrew J. Spillane, Oliver Spiro, Jonathan Spring, Johan Staaf, Peter F. Stadler, Peter Staib, Stefan G. Stark, Lucy Stebbings, Ólafur Andri Stefánsson, Oliver Stegle, Lincoln D. Stein, Alasdair Stenhouse, Chip Stewart, Stephan Stilgenbauer, Miranda D. Stobbe, Michael R. Stratton, Jonathan R. Stretch, Adam J. Struck, Joshua M. Stuart, Henk G. Stunnenberg, Hong Su, Xiaoping Su, Ren X. Sun, Stephanie Sungalee, Hana Susak, Akihiro Suzuki, Fred Sweep, Monika Szczepanowski, Holger Sültmann, Takashi Yugawa, Angela Tam, David Tamborero, Benita Kiat Tee Tan, Donghui Tan, Patrick Tan, Hiroko Tanaka, Hirokazu Taniguchi, Tomas J. Tanskanen, Maxime Tarabichi, Roy Tarnuzzer, Patrick Tarpey, Morgan L. Taschuk, Kenji Tatsuno, Simon Tavaré, Darrin F. Taylor, Amaro Taylor-Weiner, Jon W. Teague, Bin Tean Teh, Varsha Tembe, Javier Temes, Kevin Thai, Sarah P. Thayer, Nina Thiessen, Gilles Thomas, Sarah Thomas, Alan Thompson, Alastair M. Thompson, John F. Thompson, R. Houston Thompson, Heather Thorne, Leigh B. Thorne, Adrian Thorogood, Grace Tiao, Nebojsa Tijanic, Lee E. Timms, Roberto Tirabosco, Marta Tojo, Stefania Tommasi, Christopher W. Toon, Umut H. Toprak, David Torrents, Giampaolo Tortora, Jörg Tost, Yasushi Totoki, David Townend, Nadia Traficante, Isabelle Treilleux, Jean-Rémi Trotta, Lorenz H. P. Trümper, Ming Tsao, Tatsuhiko Tsunoda, Jose M. C. Tubio, Olga Tucker, Richard Turkington, Daniel J. Turner, Andrew Tutt, Masaki Ueno, Naoto T. Ueno, Christopher Umbricht, Husen M. Umer, Timothy J. Underwood, Lara Urban, Tomoko Urushidate, Tetsuo Ushiku, Liis Uusküla-Reimand, Alfonso Valencia, David J. Van Den Berg, Steven Van Laere, Peter Van Loo, Erwin G. Van Meir, Gert G. Van den Eynden, Theodorus Van der Kwast, Naveen Vasudev, Miguel Vazquez, Ravikiran Vedururu, Umadevi Veluvolu, Shankar Vembu, Lieven P. C. Verbeke, Peter Vermeulen, Clare Verrill, Alain Viari, David Vicente, Caterina Vicentini, K. Vijay Raghavan, Juris Viksna, Ricardo E. Vilain, Izar Villasante, Anne Vincent-Salomon, Tapio Visakorpi, Douglas Voet, Paresh Vyas, Ignacio Vázquez-García, Nick M. Waddell, Nicola Waddell, Claes Wadelius, Lina Wadi, Rabea Wagener, Jeremiah A. Wala, Jian Wang, Jiayin Wang, Linghua Wang, Qi Wang, Wenyi Wang, Yumeng Wang, Zhining Wang, Paul M. Waring, Hans-Jörg Warnatz, Jonathan Warrell, Anne Y. Warren, Sebastian M. Waszak, David C. Wedge, Dieter Weichenhan, Paul Weinberger, John N. Weinstein, Joachim Weischenfeldt, Daniel J. Weisenberger, Ian Welch, Michael C. Wendl, Johannes Werner, Justin P. Whalley, David A. Wheeler, Hayley C. Whitaker, Dennis Wigle, Matthew D. Wilkerson, Ashley Williams, James S. Wilmott, Gavin W. Wilson, Julie M. Wilson, Richard K. Wilson, Boris Winterhoff, Jeffrey A. Wintersinger, Maciej Wiznerowicz, Stephan Wolf, Bernice H. Wong, Tina Wong, Winghing Wong, Youngchoon Woo, Scott Wood, Bradly G. Wouters, Adam J. Wright, Derek W. Wright, Mark H. Wright, Chin-Lee Wu, Dai-Ying Wu, Guanming Wu, Jianmin Wu, Kui Wu, Yang Wu, Zhenggang Wu, Liu Xi, Tian Xia, Qian Xiang, Xiao Xiao, Rui Xing, Heng Xiong, Qinying Xu, Yanxun Xu, Hong Xue, Shinichi Yachida, Sergei Yakneen, Rui Yamaguchi, Takafumi N. Yamaguchi, Masakazu Yamamoto, Shogo Yamamoto, Hiroki Yamaue, Fan Yang, Huanming Yang, Jean Y. Yang, Liming Yang, Lixing Yang, Shanlin Yang, Tsun-Po Yang, Yang Yang, Xiaotong Yao, Marie-Laure Yaspo, Lucy Yates, Christina Yau, Chen Ye, Kai Ye, Venkata D. Yellapantula, Christopher J. Yoon, Sung-Soo Yoon, Fouad Yousif, Jun Yu, Kaixian Yu, Willie Yu, Yingyan Yu, Ke Yuan, Yuan Yuan, Denis Yuen, Takashi Yugawa, Christina K. Yung, Olga Zaikova, Jorge Zamora, Marc Zapatka, Jean C. Zenklusen, Thorsten Zenz, Nikolajs Zeps, Cheng-Zhong Zhang, Fan Zhang, Hailei Zhang, Hongwei Zhang, Hongxin Zhang, Jiashan Zhang, Jing Zhang, Junjun Zhang, Xiuqing Zhang, Xuanping Zhang, Yan Zhang, Zemin Zhang, Zhongming Zhao, Liangtao Zheng, Xiuqing Zheng, Wanding Zhou, Yong Zhou, Bin Zhu, Hongtu Zhu, Jingchun Zhu, Shida Zhu, Lihua Zou, Xueqing Zou, Anna deFazio, Nicholas van As, Carolien H. M. van Deurzen, Marc J. van de Vijver, L. van’t Veer, Christian von Mering

**Affiliations:** 1 The McDonnell Genome Institute at Washington University, St. Louis, MO 63108 USA; 2 Division of Oncology, Department of Medicine, Washington University School of Medicine, St. Louis, MO 63108 USA; 3 Alvin J. Siteman Cancer Center, Washington University School of Medicine, St. Louis, MO 63108 USA; 4 Yale School of Medicine, Yale University, New Haven, CT 06520 USA; 5 Program in Computational Biology and Bioinformatics, Yale University, New Haven, CT 06520 USA; 6 Computational Biology Program, Ontario Institute for Cancer Research, Toronto, ON M5G 0A3 Canada; 7 The Hospital for Sick Children, Toronto, ON M5G 1X8 Canada; 8 Broad Institute of MIT and Harvard, Cambridge, MA 02142 USA; 9 Biomedical Engineering, Oregon Health and Science University, Portland, OR 97239 USA; 10 Department of Mathematics, Washington University in St. Louis, St. Louis, MO 63130 USA; 11 Department of Genetics, Washington University School of Medicine, St.Louis, MO 63110 USA; 12 Human Genome Sequencing Center, Baylor College of Medicine, Houston, TX 77030 USA; 13 Department of Molecular and Human Genetics, Baylor College of Medicine, Houston, TX 77030 USA; 14 Harvard Medical School, Boston, MA 02115 USA; 15 Center for Cancer Research, Massachusetts General Hospital, Boston, MA 02114 USA; 16 Department of Pathology, Massachusetts General Hospital, Boston, MA 02114 USA; 17 Department of Computer Science, University of Toronto, Toronto, ON M5S Canada; 18 Department of Computer Science, Yale University, New Haven, CT 06520 USA; 19 Department of Molecular Biophysics and Biochemistry, Yale University, New Haven, CT 06520 USA; 20 Department of Medicine and Department of Genetics, Washington University School of Medicine, St. Louis, MO 63110 USA; 21 Department of Bioinformatics and Computational Biology, The University of Texas MD Anderson Cancer Center, Houston, TX 77030 USA; 22 Molecular and Medical Genetics, OHSU Knight Cancer Institute, Oregon Health and Science University, Portland, OR 97239 USA; 23 Castle Biosciences Inc, Friendswood, TX 77546 USA; 24 UC Santa Cruz Genomics Institute, University of California Santa Cruz, Santa Cruz, CA 95064 USA; 25 Howard Hughes Medical Institute, University of California Santa Cruz, Santa Cruz, CA 95064 USA; 26 Massachusetts General Hospital Center for Cancer Research, Charlestown, MA 02114 USA; 27 National Human Genome Research Institute, National Institutes of Health, Bethesda, MD 20894 USA; 28 Marie-Josée and Henry R. Kravis Center for Molecular Oncology, Memorial Sloan Kettering Cancer Center, New York, NY 10065 USA; 29 Ontario Institute for Cancer Research, Toronto, ON M5G 0A3 Canada; 30 Canada’s Michael Smith Genome Sciences Centre, BC Cancer, Vancouver, BC V5Z 4S6 Canada; 31 Computational Biology Program, School of Medicine, Oregon Health and Science University, Portland, OR 97239 USA; 32 DNAnexus Inc, Mountain View, CA 94040 USA; 33 Department of Systems Biology, UT MD Anderson Cancer Center, Houston, TX 77030 USA; 34 Precision Oncology, OHSU Knight Cancer Institute, Oregon Health and Science University, Portland, OR 97239 USA; 35 Computer Network Information Center, Chinese Academy of Sciences, Beijing, China; 36 Institute for Systems Biology, Seattle, WA 98109 USA; 37 Department of Biomolecular Engineering, University of California Santa Cruz, Santa Cruz, CA 95064 USA; 38 Department of Bioinformatics and Computational Biology and Department of Systems Biology, The University of Texas MD Anderson Cancer Center, Houston, TX 77030 USA; 39 Biomolecular Engineering Department, University of California Santa Cruz, Santa Cruz, CA 95064 USA; 40 School of Electronic and Information Engineering, Xi’an Jiaotong University, Xi’an, China; 41 The First Affiliated Hospital, Xi’an Jiaotong University, Xi’an, China; 42 Center for Digital Health, Berlin Institute of Health and Charitè-Universitätsmedizin Berlin, 10178 Berlin, Germany; 43 Heidelberg Center for Personalized Oncology (DKFZ-HIPO), German Cancer Research Center (DKFZ), 69120 Heidelberg, Germany; 44 Department of Medical Biophysics, University of Toronto, Toronto, ON M5S Canada; 45 Department of Human Genetics, University of California Los Angeles, Los Angeles, CA 90095 USA; 46 Department of Pharmacology, University of Toronto, Toronto, ON M5S Canada; 47 Division of Theoretical Bioinformatics, German Cancer Research Center (DKFZ), 69120 Heidelberg, Germany; 48 Institute of Pharmacy and Molecular Biotechnology and BioQuant, Heidelberg University, 69117 Heidelberg, Germany; 49 Wellcome Sanger Institute, Wellcome Genome Campus, Hinxton, CB10 1SA UK; 50 University of Texas MD Anderson Cancer Center, Houston, TX 77030 USA; 51 Department of Genetics, Informatics Institute, University of Alabama at Birmingham, Birmingham, AL 77030 USA; 52 Universitat Pompeu Fabra (UPF), 08002 Barcelona, Spain; 53 Centre for Genomic Regulation (CRG), The Barcelona Institute of Science and Technology, Barcelona, Spain; 54 Heidelberg University, 69117 Heidelberg, Germany; 55 New BIH Digital Health Center, Berlin Institute of Health (BIH) and Charité-Universitätsmedizin Berlin, 08036 Berlin, Germany; 56 BGI-Shenzhen, Shenzhen, China; 57 Barcelona Supercomputing Center (BSC), 08034 Barcelona, Spain; 58 Department Biochemistry and Molecular Biomedicine, University of Barcelona, 08007 Barcelona, Spain; 59 European Molecular Biology Laboratory, European Bioinformatics Institute (EMBL-EBI), Cambridge, CB10 1SD UK; 60 Genome Biology Unit, European Molecular Biology Laboratory (EMBL), 22607 Heidelberg, Germany; 61 CNAG-CRG, Centre for Genomic Regulation (CRG), Barcelona Institute of Science and Technology (BIST), 08036 Barcelona, Spain; 62 Vancouver Prostate Centre, Vancouver, BC V6H 3Z6 Canada; 63 Department of Urologic Sciences, University of British Columbia, Vancouver, BC V6T 1Z4 Canada; 64 Division of Life Science and Applied Genomics Center, Hong Kong University of Science and Technology, Clear Water Bay, Hong Kong, China; 65 Geneplus-Shenzhen, Shenzhen, China; 66 School of Computer Science and Technology, Xi’an Jiaotong University, Xi’an, China; 67 National Center for Tumor Diseases (NCT) Heidelberg, 69120 Heidelberg, Germany; 68 German Cancer Consortium (DKTK), 69120 Heidelberg, Germany; 69 Genome Integration Data Center, Syntekabio, Inc, Daejeon, South Korea; 70 Department of Biochemistry, College of Medicine, Ewha Womans University, Seoul, South Korea; 71 Biobyte solutions GmbH, 69126 Heidelberg, Germany; 72 Massachusetts General Hospital, Boston, MA 02114 USA; 73 Department of Molecular Medicine (MOMA), Aarhus University Hospital, 8200 Aarhus N, Denmark; 74 Institute of Medical Genetics and Applied Genomics, University of Tübingen, 72074 Tübingen, Germany; 75 Bioinformatics Research Centre (BiRC), Aarhus University, 8000 Aarhus, Denmark; 76 Genome Informatics Program, Ontario Institute for Cancer Research, Toronto, ON M5G 0A3 Canada; 77 Department of Radiation Oncology, University of California San Francisco, San Francisco, CA 94110 USA; 78 Simon Fraser University, Burnaby, BC V5A 1S6 Canada; 79 Indiana University, Bloomington, IN 47405 USA; 80 Bioinformatics and Omics Data Analytics, German Cancer Research Center (DKFZ), 69120 Heidelberg, Germany; 81 Institució Catalana de Recerca i Estudis Avançats (ICREA), Barcelona, Spain; 82 Department of Medical Oncology, Dana-Farber Cancer Institute, Boston, MA 02215 USA; 83 Finsen Laboratory and Biotech Research and Innovation Centre (BRIC), University of Copenhagen, 1165 Copenhagen, Denmark; 84 Department of Urology, Charité Universitätsmedizin Berlin, 10117 Berlin, Germany; 85 Department of Biological Oceanography, Leibniz Institute of Baltic Sea Research, 18119 Rostock, Germany; 86 Department of Epidemiology and Biostatistics, Memorial Sloan Kettering Cancer Center, New York, NY 10065 USA; 87 Applied Tumor Genomics Research Program, Research Programs Unit, University of Helsinki, 00100 Helsinki, Finland; 88 Memorial Sloan Kettering Cancer Center, New York, NY 10065 USA; 89 Genome Science Division, Research Center for Advanced Science and Technology, University of Tokyo, Tokyo, 113-8654 Japan; 90 Department of Surgery, University of Chicago, Chicago, IL 60637 USA; 91 Department of Surgery, Division of Hepatobiliary and Pancreatic Surgery, School of Medicine, Keimyung University Dongsan Medical Center, Daegu, South Korea; 92 Department of Oncology, Gil Medical Center, Gachon University, Incheon, South Korea; 93 Hiroshima University, Hiroshima, 739-8511 Japan; 94 King Faisal Specialist Hospital and Research Centre, Al Maather, Riyadh, Saudi Arabia; 95 Bioinformatics Unit, Spanish National Cancer Research Centre (CNIO), 28029 Madrid, Spain; 96 Bioinformatics Core Facility, University Medical Center Hamburg, 20251 Hamburg, Germany; 97 Heinrich Pette Institute, Leibniz Institute for Experimental Virology, 20251 Hamburg, Germany; 98 Ontario Tumour Bank, Ontario Institute for Cancer Research, Toronto, ON M5G 0A3 Canada; 99 Department of Pathology, The University of Texas MD Anderson Cancer Center, Houston, TX 77030 USA; 100 Laboratory of Pathology, Center for Cancer Research, National Cancer Institute, Bethesda, MD 20814 USA; 101 Department of Cellular and Molecular Medicine and Department of Bioengineering, University of California San Diego, La Jolla, CA 92093 USA; 102 UC San Diego Moores Cancer Center, San Diego, CA 92037 USA; 103 Sir Peter MacCallum Department of Oncology, Peter MacCallum Cancer Centre, University of Melbourne, Melbourne, VIC 3010 Australia; 104 Centre for Research in Molecular Medicine and Chronic Diseases (CiMUS), Universidade de Santiago de Compostela, 15705 Santiago de Compostela, Spain; 105 Department of Zoology, Genetics and Physical Anthropology, (CiMUS), Universidade de Santiago de Compostela, 15705 Santiago de Compostela, Spain; 106 The Biomedical Research Centre (CINBIO), Universidade de Vigo, 36310 Vigo, Spain; 107 Royal National Orthopaedic Hospital-Bolsover, London, 36310 UK; 108 Department of Genomic Medicine, The University of Texas MD Anderson Cancer Center, Houston, TX 77030 USA; 109 Quantitative and Computational Biosciences Graduate Program, Baylor College of Medicine, Houston, TX 77030 USA; 110 The Jackson Laboratory for Genomic Medicine, Farmington, CT 06032 USA; 111 Institute of Human Genetics, Christian-Albrechts-University, 24118 Kiel, Germany; 112 Institute of Human Genetics, Ulm University and Ulm University Medical Center, 89081 Ulm, Germany; 113 Queensland Centre for Medical Genomics, Institute for Molecular Bioscience, University of Queensland, St. Lucia, Brisbane, QLD 4072 Australia; 114 Salford Royal NHS Foundation Trust, Salford, M6 8HD UK; 115 Department of Surgery, Pancreas Institute, University and Hospital Trust of Verona, 37129 Verona, Italy; 116 Department of Molecular Oncology, BC Cancer Research Centre, Vancouver, BC V5Z 1L3 Canada; 117 University College London, London, WC1E 6BT UK; 118 Division of Cancer Genomics, National Cancer Center Research Institute, National Cancer Center, Tokyo, 104-0045 Japan; 119 DLR Project Management Agency, Bonn, Germany; 120 Tokyo Women’s Medical University, Tokyo, 162-8666 Japan; 121 Center for Molecular Oncology, Memorial Sloan Kettering Cancer Center, New York, NY 10065 USA; 122 Los Alamos National Laboratory, Los Alamos, NM 87545 USA; 123 Department of Pathology, University Health Network, Toronto General Hospital, Toronto, ON M5G 2C4 Canada; 124 Nottingham University Hospitals NHS Trust, Nottingham, NG5 1PB UK; 125 Epigenomics and Cancer Risk Factors, German Cancer Research Center (DKFZ), 69120 Heidelberg, Germany; 126 Department of Molecular Genetics, University of Toronto, Toronto, ON M5S Canada; 127 Vector Institute, Toronto, ON M5G 1M1 Canada; 128 Hematopathology Section, Institute of Pathology, Christian-Albrechts-University, Kiel, 24118 Germany; 129 Department of Pathology and Laboratory Medicine, School of Medicine, University of North Carolina at Chapel Hill, Chapel Hill, NC 27599 USA; 130 Department of Cancer Genetics, Institute for Cancer Research, Oslo University Hospital, The Norwegian Radium Hospital, 0450 Oslo, Norway; 131 Pathology, Hospital Clinic, Institut d’Investigacions Biomèdiques August Pi i Sunyer (IDIBAPS), University of Barcelona, 08007 Barcelona, Spain; 132 Department of Veterinary Medicine, Transmissible Cancer Group, University of Cambridge, Cambridge, CB2 1TN UK; 133 Wolfson Wohl Cancer Research Centre, Institute of Cancer Sciences, University of Glasgow, Glasgow, G12 8QQ UK; 134 Lineberger Comprehensive Cancer Center, University of North Carolina at Chapel Hill, Chapel Hill, NC 27599 USA; 135 Dana-Farber/Boston Children’s Cancer and Blood Disorders Center, Boston, MA 02115 USA; 136 Department of Pediatrics, Harvard Medical School, Boston, MA 02115 USA; 137 Leeds Institute of Medical Research at St. James’s, University of Leeds, St. James’s University Hospital, Leeds, LS2 9JT UK; 138 Department of Pathology and Diagnostics, University and Hospital Trust of Verona, 37129 Verona, Italy; 139 Department of Surgery, Princess Alexandra Hospital, Brisbane, QLD 4102 Australia; 140 Surgical Oncology Group, Diamantina Institute, University of Queensland, Brisbane, QLD 4072 Australia; 141 Department of Population and Quantitative Health Sciences, Case Western Reserve University School of Medicine, Cleveland, OH 44106 USA; 142 Research Health Analytics and Informatics, University Hospitals Cleveland Medical Center, Cleveland, OH 44106 USA; 143 Gloucester Royal Hospital, Gloucester, GL1 3NN UK; 144 Diagnostic Development, Ontario Institute for Cancer Research, Toronto, ON M5G 0A3 Canada; 145 Arnie Charbonneau Cancer Institute, University of Calgary, Calgary, AB T2N 1N4 Canada; 146 Departments of Surgery and Oncology, University of Calgary, Calgary, AB T2N 1N4 Canada; 147 Department of Pathology, Oslo University Hospital, The Norwegian Radium Hospital, Oslo, Norway; 148 PanCuRx Translational Research Initiative, Ontario Institute for Cancer Research, Toronto, ON M5G 0A3 Canada; 149 Department of Oncology, Sidney Kimmel Comprehensive Cancer Center at Johns Hopkins University School of Medicine, Baltimore, MD 21231 USA; 150 University Hospital Southampton NHS Foundation Trust, Southampton, SO16 6YD UK; 151 Royal Stoke University Hospital, Stoke-on-Trent, ST4 6QG UK; 152 Genome Sequence Informatics, Ontario Institute for Cancer Research, Toronto, ON M5G 0A3 Canada; 153 Human Longevity Inc, San Diego, CA M5G 0A3 USA; 154 Olivia Newton-John Cancer Research Institute, La Trobe University, Heidelberg, VIC 3086 Australia; 155 Genome Canada, Ottawa, ON K2P 1P1 Canada; 156 Buck Institute for Research on Aging, Novato, CA 94945 USA; 157 Duke University Medical Center, Durham, NC 27710 USA; 158 Department of Human Genetics, Hannover Medical School, 30625 Hannover, Germany; 159 Center for Bioinformatics and Functional Genomics, Cedars-Sinai Medical Center, Los Angeles, CA 90048 USA; 160 Department of Biomedical Sciences, Cedars-Sinai Medical Center, Los Angeles, CA 90048 USA; 161 The Hebrew University Faculty of Medicine, Jerusalem, 9112102 Israel; 162 Barts Cancer Institute, Barts and the London School of Medicine and Dentistry, Queen Mary University of London, London, E1 4NS UK; 163 Department of Computer Science, Bioinformatics Group, University of Leipzig, 04109 Leipzig, Germany; 164 Interdisciplinary Center for Bioinformatics, University of Leipzig, 04109 Leipzig, Germany; 165 Transcriptome Bioinformatics, LIFE Research Center for Civilization Diseases, University of Leipzig, 04109 Leipzig, Germany; 166 USC Norris Comprehensive Cancer Center, University of Southern California, Los Angeles, CA 90007 USA; 167 Department of Diagnostics and Public Health, University and Hospital Trust of Verona, Verona, 37129 Italy; 168 Department of Mathematics, Aarhus University, 8000 Aarhus, Denmark; 169 Instituto Carlos Slim de la Salud, Mexico City, Mexico; 170 Cancer Division, Garvan Institute of Medical Research, Kinghorn Cancer Centre, University of New South Wales (UNSW Sydney), Sydney, NSW 2052 Australia; 171 South Western Sydney Clinical School, Faculty of Medicine, University of New South Wales (UNSW Sydney), Liverpool, NSW 2052 Australia; 172 West of Scotland Pancreatic Unit, Glasgow Royal Infirmary, Glasgow, G4 0SF UK; 173 The Preston Robert Tisch Brain Tumor Center, Duke University Medical Center, Durham, NC 27710 USA; 174 National Institute of Biomedical Genomics, Kalyani, West Bengal 741251 India; 175 Institute of Clinical Medicine and Institute of Oral Biology, University of Oslo, 0315 Oslo, Norway; 176 University of North Carolina at Chapel Hill, Chapel Hill, NC 27599 USA; 177 ARC-Net Centre for Applied Research on Cancer, University and Hospital Trust of Verona, Verona, 0315 Italy; 178 The Institute of Cancer Research, London, SM2 5NG UK; 179 Centre for Computational Biology, Duke-NUS Medical School, Singapore, 169857 Singapore; 180 Programme in Cancer and Stem Cell Biology, Duke-NUS Medical School, Singapore, 169857 Singapore; 181 Division of Oncology and Pathology, Department of Clinical Sciences Lund, Lund University, Lund, Sweden; 182 Department of Pediatric Oncology, Hematology and Clinical Immunology, Heinrich-Heine-University, 40225 Düsseldorf, Germany; 183 Laboratory for Medical Science Mathematics, RIKEN Center for Integrative Medical Sciences, Yokohama, Japan; 184 RIKEN Center for Integrative Medical Sciences, Yokohama, Japan; 185 Department of Internal Medicine/Hematology, Friedrich-Ebert-Hospital, Neumünster, Germany; 186 Departments of Dermatology and Pathology, Yale University, New Haven, CT 06520 USA; 187 Radcliffe Department of Medicine, University of Oxford, Oxford, OX1 2JD UK; 188 Canadian Center for Computational Genomics, McGill University, Montreal, QC H3A 0G4 Canada; 189 Department of Human Genetics, McGill University, Montreal, QC H3A 0G4 Canada; 190 Faculty of Medicine and Health Technology, Tampere University and Tays Cancer Center, Tampere University Hospital, 33520 Tampere, Finland; 191 Haematology, Leeds Teaching Hospitals NHS Trust, Leeds, LS1 3EX UK; 192 Translational Research and Innovation, Centre Léon Bérard, Lyon, France; 193 Fox Chase Cancer Center, Philadelphia, PA 19111 USA; 194 International Agency for Research on Cancer, World Health Organization, Lyon, France; 195 Earlham Institute, Norwich, NR4 7UZ UK; 196 Norwich Medical School, University of East Anglia, Norwich, NR4 7TJ UK; 197 Department of Molecular Biology, Faculty of Science, Radboud Institute for Molecular Life Sciences, Radboud University, Nijmegen, HB 6525 XZ The Netherlands; 198 CRUK Manchester Institute and Centre, Manchester, M20 4GJ UK; 199 Department of Radiation Oncology, University of Toronto, Toronto, ON M5S Canada; 200 Division of Cancer Sciences, Manchester Cancer Research Centre, University of Manchester, Manchester, M13 9PL UK; 201 Radiation Medicine Program, Princess Margaret Cancer Centre, Toronto, ON M5G 2C1 Canada; 202 Department of Pathology, Brigham and Women’s Hospital, Harvard Medical School, Boston, MA 02115 USA; 203 Department of Surgery, Division of Thoracic Surgery, The Johns Hopkins University School of Medicine, Baltimore, MD 21205 USA; 204 Division of Molecular Pathology, The Netherlands Cancer Institute, Oncode Institute, Amsterdam, CX 1066 The Netherlands; 205 Division of Applied Bioinformatics, German Cancer Research Center (DKFZ), 69120 Heidelberg, Germany; 206 German Cancer Genome Consortium (DKTK), Heidelberg, Germany; 207 Center for Biological Sequence Analysis, Department of Bio and Health Informatics, Technical University of Denmark, 2800 Lyngby, Denmark; 208 Novo Nordisk Foundation Center for Protein Research, University of Copenhagen, Copenhagen, 1165 Denmark; 209 Institute for Molecular Bioscience, University of Queensland, St. Lucia, Brisbane, QLD 4072 Australia; 210 Federal Ministry of Education and Research, Berlin, Germany; 211 Melanoma Institute Australia, University of Sydney, Sydney, NSW 2006 Australia; 212 Pediatric Hematology and Oncology, University Hospital Muenster, 48149 Muenster, Germany; 213 Department of Pathology, Johns Hopkins University School of Medicine, Baltimore, MD 21205 USA; 214 McKusick-Nathans Institute of Genetic Medicine, Sidney Kimmel Comprehensive Cancer Center at Johns Hopkins University School of Medicine, Baltimore, MD 21231 USA; 215 Foundation Medicine, Inc, Cambridge, MA 02141 USA; 216 Department of Biomedical Data Science, Stanford University School of Medicine, Stanford, CA 94305 USA; 217 Department of Genetics, Stanford University School of Medicine, Stanford, CA 94305 USA; 218 Bakar Computational Health Sciences Institute and Department of Pediatrics, University of California, San Francisco, CA 94110 USA; 219 Institute of Clinical Medicine, Faculty of Medicine, University of Oslo, Oslo, 0315 Norway; 220 National Cancer Institute, National Institutes of Health, Bethesda, MD 20892 USA; 221 Royal Marsden NHS Foundation Trust, London and Sutton, Sutton, SM2 5PT UK; 222 Department of Oncology, University of Cambridge, Cambridge, CB2 1TN UK; 223 Li Ka Shing Centre, Cancer Research UK Cambridge Institute, University of Cambridge, Cambridge, CB2 1TN UK; 224 Institut Gustave Roussy, 94800 Villejuif, France; 225 Department of Haematology, University of Cambridge, Cambridge, CB2 1TN UK; 226 Anatomia Patológica, Hospital Clinic, Institut d’Investigacions Biomèdiques August Pi i Sunyer (IDIBAPS), University of Barcelona, 08007 Barcelona, Spain; 227 Spanish Ministry of Science and Innovation, Madrid, Spain; 228 University of Michigan Comprehensive Cancer Center, Ann Arbor, MI 48109 USA; 229 Department for BioMedical Research, University of Bern, 3012 Bern, Switzerland; 230 Department of Medical Oncology, Inselspital, University Hospital and University of Bern, 3012 Bern, Switzerland; 231 Graduate School for Cellular and Biomedical Sciences, University of Bern, 3012 Bern, Switzerland; 232 University of Pavia, 27100 Pavia, Italy; 233 University of Alabama at Birmingham, Birmingham, AL 35294 USA; 234 UHN Program in BioSpecimen Sciences, Toronto General Hospital, Toronto, ON M5G 2C4 Canada; 235 Department of Urology, Icahn School of Medicine at Mount Sinai, New York, NY 10029 USA; 236 Centre for Law and Genetics, University of Tasmania, Sandy Bay Campus, Hobart, TAS 7005 Australia; 237 Faculty of Biosciences, Heidelberg University, 69117 Heidelberg, Germany; 238 Department of Biochemistry, Microbiology and Immunology, Faculty of Medicine, University of Ottawa, Ottawa, ON K1N 6N5 Canada; 239 Division of Anatomic Pathology, Mayo Clinic, Rochester, MN 55905 USA; 240 Division of Cancer Epidemiology and Genetics, National Cancer Institute, National Institutes of Health, Bethesda, MD 20892 USA; 241 Illawarra Shoalhaven Local Health District L3 Illawarra Cancer Care Centre, Wollongong Hospital, Wollongong, NSW 2500 Australia; 242 BioForA, French National Institute for Agriculture, Food, and Environment (INRAE), ONF, Orléans, France; 243 Department of Biostatistics, Bloomberg School of Public Health, Johns Hopkins University, Baltimore, MD 21218 USA; 244 University of California San Diego, San Diego, CA 92093 USA; 245 Division of Experimental Pathology, Mayo Clinic, Rochester, MN 55905 USA; 246 Centre for Cancer Research, The Westmead Institute for Medical Research, University of Sydney, Sydney, NSW 2006 Australia; 247 Department of Gynaecological Oncology, Westmead Hospital, Sydney, NSW 2145 Australia; 248 PDXen Biosystems Inc, Seoul, South Korea; 249 Korea Advanced Institute of Science and Technology, Daejeon, South Korea; 250 Electronics and Telecommunications Research Institute, Daejeon, South Korea; 251 Institut National du Cancer (INCA), Boulogne-Billancourt, France; 252 Division of Medical Oncology, National Cancer Centre, Singapore, 169610 Singapore; 253 Medical Oncology, University and Hospital Trust of Verona, Verona, 37129 Italy; 254 Department of Pediatrics, University Hospital Schleswig-Holstein, 23562 Kiel, Germany; 255 Hepatobiliary/Pancreatic Surgical Oncology Program, University Health Network, Toronto, ON M5G 1L7 Canada; 256 School of Biological Sciences, University of Auckland, Auckland, 1010 New Zealand; 257 Department of Surgery, University of Melbourne, Parkville, VIC 3010 Australia; 258 The Murdoch Children’s Research Institute, Royal Children’s Hospital, Parkville, VIC 3052 Australia; 259 Walter and Eliza Hall Institute, Parkville, VIC 3052 Australia; 260 Lunenfeld-Tanenbaum Research Institute, Mount Sinai Hospital, Toronto, ON M5G 1X5 Canada; 261 University of East Anglia, Norwich, NR4 7TJ UK; 262 Norfolk and Norwich University Hospital NHS Trust, Norwich, NR4 7UY UK; 263 Victorian Institute of Forensic Medicine, Southbank, VIC 3006 Australia; 264 Department of Biomedical Informatics, Harvard Medical School, Boston, MA 02115 USA; 265 Department of Chemistry, Centre for Molecular Science Informatics, University of Cambridge, Cambridge, CB2 1TN UK; 266 Ludwig Center at Harvard Medical School, Boston, MA 02115 USA; 267 Peter MacCallum Cancer Centre, University of Melbourne, Melbourne, VIC 3010 Australia; 268 Physics Division, Optimization and Systems Biology Lab, Massachusetts General Hospital, Boston, MA 02114 USA; 269 Department of Medicine, Baylor College of Medicine, Houston, TX 77030 USA; 270 University of Cologne, 50923 Cologne, Germany; 271 International Genomics Consortium, Phoenix, AZ 85004 USA; 272 Genomics Research Program, Ontario Institute for Cancer Research, Toronto, ON M5G 0A3 Canada; 273 Barking Havering and Redbridge University Hospitals NHS Trust, Romford, UK; 274 Children’s Hospital at Westmead, University of Sydney, Sydney, NSW 2006 Australia; 275 Department of Medicine, Section of Endocrinology, University and Hospital Trust of Verona, 37129 Verona, Italy; 276 Computational Biology Center, Memorial Sloan Kettering Cancer Center, New York, NY 10065 USA; 277 Department of Biology, , ETH Zurich, Zürich, Switzerland; 278 Department of Computer Science, ETH Zurich, Zurich, Switzerland; 279 SIB Swiss Institute of Bioinformatics, Lausanne, Switzerland; 280 Weill Cornell Medical College, New York, NY 10021 USA; 281 Academic Department of Medical Genetics, University of Cambridge, Addenbrooke’s Hospital, Cambridge, CB2 1TN UK; 282 MRC Cancer Unit, University of Cambridge, Cambridge, CB2 1TN UK; 283 Departments of Pediatrics and Genetics, University of North Carolina at Chapel Hill, Chapel Hill, NC 27599 USA; 284 Seven Bridges Genomics, Charlestown, MA 02129 USA; 285 Annai Systems, Inc, Carlsbad, CA 92013 USA; 286 Department of Pathology, General Hospital of Treviso, Department of Medicine, University of Padua, 35122 Treviso, Italy; 287 Department of Computational Biology, University of Lausanne, 1015 Lausanne, Switzerland; 288 Department of Genetic Medicine and Development, University of Geneva Medical School, 1205 CH Geneva, Switzerland; 289 Swiss Institute of Bioinformatics, University of Geneva, 1205 CH Geneva, Switzerland; 290 The Francis Crick Institute, London, NW1 1AT UK; 291 University of Leuven, 3000 Leuven, Belgium; 292 Computational and Systems Biology, Genome Institute of Singapore, Singapore, 138672 Singapore; 293 School of Computing, National University of Singapore, Singapore, 119077 Singapore; 294 Big Data Institute, Li Ka Shing Centre, University of Oxford, Oxford, OX1 2JD UK; 295 The Edward S. Rogers Sr. Department of Electrical and Computer Engineering, University of Toronto, Toronto, ON M5S Canada; 296 Breast Cancer Translational Research Laboratory JC Heuson, Institut Jules Bordet, 1000 Brussels, Belgium; 297 Department of Oncology, Laboratory for Translational Breast Cancer Research, KU Leuven, 3000 Leuven, Belgium; 298 Institute for Research in Biomedicine (IRB Barcelona), The Barcelona Institute of Science and Technology, 08036 Barcelona, Spain; 299 Research Program on Biomedical Informatics, Universitat Pompeu Fabra, 08002 Barcelona, Spain; 300 Division of Medical Oncology, Princess Margaret Cancer Centre, Toronto, ON M5G 2C1 Canada; 301 Department of Physiology and Biophysics, Weill Cornell Medicine, New York, NY 10065 USA; 302 Institute for Computational Biomedicine, Weill Cornell Medicine, New York, NY 10065 USA; 303 Department of Pathology, UPMC Shadyside, Pittsburgh, PA 15232 USA; 304 Independent Consultant, Wellesley, USA; 305 Department of Cell and Molecular Biology, Science for Life Laboratory, Uppsala University, 752 36 Uppsala, Sweden; 306 Hefei University of Technology, Anhui, China; 307 Translational Cancer Research Unit, GZA Hospitals St.-Augustinus, Center for Oncological Research, Faculty of Medicine and Health Sciences, University of Antwerp, Antwerp, 2000 Belgium; 308 University of Pennsylvania, Philadelphia, PA 19104 USA; 309 The Wellcome Trust, London, NW1 2BE UK; 310 Department of Pathology, Queen Elizabeth University Hospital, Glasgow, G51 4TF UK; 311 Department of Genetics and Computational Biology, QIMR Berghofer Medical Research Institute, Brisbane, QLD 4006 Australia; 312 Department of Oncology, Centre for Cancer Genetic Epidemiology, University of Cambridge, Cambridge, CB2 1TN UK; 313 Department of Public Health and Primary Care, Centre for Cancer Genetic Epidemiology, University of Cambridge, Cambridge, CB2 1TN UK; 314 Prostate Cancer Canada, Toronto, ON M5C 1M1 Canada; 315 University of Cambridge, Cambridge, CB2 1TN UK; 316 Department of Laboratory Medicine, Translational Cancer Research, Lund University Cancer Center at Medicon Village, Lund University, Lund, Sweden; 317 CIBER Epidemiología y Salud Pública (CIBERESP), Madrid, Spain; 318 Research Group on Statistics, Econometrics and Health (GRECS), UdG, Barcelona, Spain; 319 Quantitative Genomics Laboratories (qGenomics), Barcelona, Spain; 320 Icelandic Cancer Registry, Icelandic Cancer Society, Reykjavik, Iceland; 321 State Key Laboratory of Cancer Biology, and Xijing Hospital of Digestive Diseases, Fourth Military Medical University, Shaanxi, China; 322 Department of Medicine (DIMED), Surgical Pathology Unit, University of Padua, 35122 Padua, Italy; 323 Department of Cancer Biology, Dana-Farber Cancer Institute, Boston, MA 02215 USA; 324 Rigshospitalet, Copenhagen, Denmark; 325 Center for Cancer Genomics, National Cancer Institute, National Institutes of Health, Bethesda, MD 20814 USA; 326 Department of Biochemistry and Molecular Medicine, University of Montreal, Montreal, QC H3T 1J4 Canada; 327 Australian Institute of Tropical Health and Medicine, James Cook University, Douglas, QLD 4811 Australia; 328 Department of Neuro-Oncology, Istituto Neurologico Besta, Milano, Italy; 329 Bioplatforms Australia, North Ryde, NSW Australia; 330 Department of Pathology (Research), University College London Cancer Institute, London, WC1E 6DD UK; 331 Department of Surgical Oncology, Princess Margaret Cancer Centre, Toronto, ON M5G 2C1 Canada; 332 Department of Medical Oncology, Josephine Nefkens Institute and Cancer Genomics Centre, Erasmus Medical Center, 3015 GD Rotterdam, The Netherlands; 333 The University of Queensland Thoracic Research Centre, The Prince Charles Hospital, Brisbane, QLD 4032 Australia; 334 CIBIO/InBIO-Research Center in Biodiversity and Genetic Resources, Universidade do Porto, 4099-002 Vairão, Portugal; 335 HCA Laboratories, London, UK; 336 University of Liverpool, Liverpool, L69 3BX UK; 337 The Azrieli Faculty of Medicine, Bar-Ilan University, Safed, 5290002 Israel; 338 Department of Neurosurgery, University of Florida, Gainesville, FL 32611 USA; 339 Department of Pathology, Graduate School of Medicine, University of Tokyo, Tokyo, 113-8654 Japan; 340 National Genotyping Center, Institute of Biomedical Sciences, Academia Sinica, Taipei, Taiwan; 341 University of Milano Bicocca, Monza, 20126 Italy; 342 Department of Pathology, Oslo University Hospital Ulleval, 0450 Oslo, Norway; 343 Center for Biomedical Informatics, Harvard Medical School, Boston, MA 02115 USA; 344 Office of Cancer Genomics, National Cancer Institute, National Institutes of Health, Bethesda, MD 20892 USA; 345 Cancer Epigenomics, German Cancer Research Center (DKFZ), 69120 Heidelberg, Germany; 346 Department of Cancer Biology, The University of Texas MD Anderson Cancer Center, Houston, TX 77030 USA; 347 Department of Surgical Oncology, The University of Texas MD Anderson Cancer Center, Houston, TX 77030 USA; 348 Department of Pathology, Memorial Sloan Kettering Cancer Center, New York, NY 10065 USA; 349 Division of Gastroenterology and Hepatology, Mayo Clinic, Rochester, MN 55905 USA; 350 University of Sydney, Sydney, NSW 2006 Australia; 351 University of Oxford, Oxford, OX1 2JD UK; 352 Cambridge University Hospitals NHS Foundation Trust, Cambridge, CB2 0QQ UK; 353 Department of Surgery, Academic Urology Group, University of Cambridge, Cambridge, CB2 0QQ UK; 354 Department of Medicine II, University of Würzburg, 97070 Wuerzburg, Germany; 355 Sylvester Comprehensive Cancer Center, University of Miami, Miami, FL 33146 USA; 356 Institut Hospital del Mar d’Investigacions Mèdiques (IMIM), Barcelona, Spain; 357 Genome Integrity and Structural Biology Laboratory, National Institute of Environmental Health Sciences (NIEHS), Durham, NC 27709 USA; 358 St. Thomas’s Hospital, London, SE1 7EH UK; 359 Osaka International Cancer Center, Osaka, Japan; 360 Department of Pathology, Skåne University Hospital, Lund University, Lund, Sweden; 361 Department of Medical Oncology, Beatson West of Scotland Cancer Centre, Glasgow, G12 0YN UK; 362 Centre for Cancer Research, Victorian Comprehensive Cancer Centre, University of Melbourne, Melbourne, VIC 3010 Australia; 363 Department of Medicine, Section of Hematology/Oncology, University of Chicago, Chicago, IL 60637 USA; 364 German Center for Infection Research (DZIF), Partner Site Hamburg-Borstel-Lübeck-Riems, Hamburg, Germany; 365 Department of Biotechnology, Ministry of Science and Technology, Government of India, New Delhi, Delhi 110016 India; 366 National Cancer Centre Singapore, Singapore, 169610 Singapore; 367 Brandeis University, Waltham, MA 02453 USA; 368 Department of Internal Medicine, Stanford University, Stanford, CA 94305 USA; 369 The University of Texas Health Science Center at Houston, Houston, TX 77030 USA; 370 Imperial College NHS Trust, Imperial College, London, INY SW7 2BU UK; 371 Senckenberg Institute of Pathology, University of Frankfurt Medical School, 60323 Frankfurt, Germany; 372 Department of Medicine, Division of Biomedical Informatics, UC San Diego School of Medicine, San Diego, CA 92093 USA; 373 Center for Precision Health, School of Biomedical Informatics, The University of Texas Health Science Center, Houston, TX 77030 USA; 374 Oxford Nanopore Technologies, New York, NY OX4 4DQ USA; 375 Institute of Medical Science, University of Tokyo, Tokyo, 113-8654 Japan; 376 Wakayama Medical University, Wakayama, 641-8510 Japan; 377 Department of Internal Medicine, Division of Medical Oncology, Lineberger Comprehensive Cancer Center, University of North Carolina at Chapel Hill, Chapel Hill, NC 27599 USA; 378 University of Tennessee Health Science Center for Cancer Research, Memphis, TN 38163 USA; 379 Department of Histopathology, Salford Royal NHS Foundation Trust, Salford, M6 8HD UK; 380 Faculty of Biology, Medicine and Health, University of Manchester, Manchester, M13 9PL UK; 381 Peking University, 100871 Beijing, China; 382 Children’s Hospital of Philadelphia, Philadelphia, PA 19104 USA; 383 Karolinska Institute, 171 77 Stockholm, Sweden; 384 The Donnelly Centre, University of Toronto, Toronto, ON M5S Canada; 385 Department of Medical Genetics, College of Medicine, Hallym University, Chuncheon, South Korea; 386 Department of Experimental and Health Sciences, Institute of Evolutionary Biology (UPF-CSIC), Universitat Pompeu Fabra, 08002 Barcelona, Spain; 387 Health Data Science Unit, University Clinics, Heidelberg, Germany; 388 Hokkaido University, Sapporo, 060-0808 Japan; 389 Department of Pathology and Clinical Laboratory, National Cancer Center Hospital, Tokyo, 104-0045 Japan; 390 Department of Genetics, University of North Carolina at Chapel Hill, Chapel Hill, NC 27599 USA; 391 Computational Biology, Leibniz Institute on Aging-Fritz Lipmann Institute (FLI), 07745 Jena, Germany; 392 University of Melbourne Centre for Cancer Research, Melbourne, VIC 3010 Australia; 393 University of Nebraska Medical Center, Omaha, NE 68198 USA; 394 Syntekabio Inc, Daejeon, South Korea; 395 Department of Pathology, Academic Medical Center, Amsterdam, 1105 AZ The Netherlands; 396 China National GeneBank-Shenzhen, Shenzhen, China; 397 Division of Molecular Genetics, German Cancer Research Center (DKFZ), 69120 Heidelberg, Germany; 398 Icahn School of Medicine at Mount Sinai, New York, NY 10029 USA; 399 AbbVie, North Chicago, IL 60064 USA; 400 Institute of Pathology, Charité – University Medicine Berlin, Berlin, Germany; 401 Centre for Translational and Applied Genomics, British Columbia Cancer Agency, Vancouver, BC V8R 6V5 Canada; 402 Edinburgh Royal Infirmary, Edinburgh, EH16 4SA UK; 403 Berlin Institute for Medical Systems Biology, Max Delbrück Center for Molecular Medicine, 13125 Berlin, Germany; 404 Department of Pediatric Immunology, Hematology and Oncology, University Hospital, Heidelberg, 69120 Heidelberg, Germany; 405 German Cancer Research Center (DKFZ), 69120 Heidelberg, Germany; 406 Heidelberg Institute for Stem Cell Technology and Experimental Medicine (HI-STEM), 69120 Heidelberg, Germany; 407 Institute for Computational Biomedicine, Weill Cornell Medical College, New York, NY 10021 USA; 408 New York Genome Center, New York, NY 10013 USA; 409 Department of Urology, James Buchanan Brady Urological Institute, Johns Hopkins University School of Medicine, Baltimore, MD 21205 USA; 410 Department of Preventive Medicine, Graduate School of Medicine, The University of Tokyo, Tokyo, 113-8654 Japan; 411 Department of Molecular and Cellular Biology, Baylor College of Medicine, Houston, TX 77030 USA; 412 Department of Pathology and Immunology, Baylor College of Medicine, Houston, TX 77030 USA; 413 Michael E. DeBakey Veterans Affairs Medical Center, Houston, TX 77030 USA; 414 Technical University of Denmark, 2800 Lyngby, Denmark; 415 Department of Pathology, College of Medicine, Hanyang University, Seoul, South Korea; 416 Academic Unit of Surgery, School of Medicine, College of Medical, Veterinary and Life Sciences, University of Glasgow, Glasgow Royal Infirmary, Glasgow, G12 8QQ UK; 417 Department of Pathology, Asan Medical Center, College of Medicine, Ulsan University, Songpa-gu, Seoul, South Korea; 418 Science Writer, Garrett Park, MD USA; 419 International Cancer Genome Consortium (ICGC)/ICGC Accelerating Research in Genomic Oncology (ARGO) Secretariat, Ontario Institute for Cancer Research, Toronto, ON M5G 0A3 Canada; 420 University of Ljubljana, 1000 Ljubljana, Slovenia; 421 Department of Public Health Sciences, University of Chicago, Chicago, IL 60637 USA; 422 Research Institute, NorthShore University HealthSystem, Evanston, IL 60201 USA; 423 Department for Biomedical Research, University of Bern, Bern, 3012 Switzerland; 424 Centre of Genomics and Policy, McGill University and Génome Québec Innovation Centre, Montreal, QC H3A 0G4 Canada; 425 Carolina Center for Genome Sciences, University of North Carolina at Chapel Hill, Chapel Hill, NC 27599 USA; 426 Hopp Children’s Cancer Center (KiTZ), Heidelberg, Germany; 427 Pediatric Glioma Research Group, German Cancer Research Center (DKFZ), 69120 Heidelberg, Germany; 428 Cancer Research UK, London, NG34 7SY UK; 429 Indivumed GmbH, Hamburg, Germany; 430 University Hospital Zurich, 8091 Zurich, Switzerland; 431 Clinical Bioinformatics, Swiss Institute of Bioinformatics, 1015 Geneva, Switzerland; 432 Institute for Pathology and Molecular Pathology, University Hospital Zurich, 8091 Zurich, Switzerland; 433 Institute of Molecular Life Sciences, University of Zurich, 8091 Zurich, Switzerland; 434 MRC Human Genetics Unit, MRC IGMM, University of Edinburgh, Edinburgh, EH8 9YL UK; 435 Women’s Cancer Program at the Samuel Oschin Comprehensive Cancer Institute, Cedars-Sinai Medical Center, Los Angeles, CA 90048 USA; 436 Department of Biology, Bioinformatics Group, Division of Molecular Biology, Faculty of Science, University of Zagreb, 10000 Zagreb, Croatia; 437 Department for Internal Medicine II, University Hospital Schleswig-Holstein, 23562 Kiel, Germany; 438 Genetics and Molecular Pathology, SA Pathology, Adelaide, SA 5011 Australia; 439 Department of Gastric Surgery, National Cancer Center Hospital, Tokyo, Japan; 440 Department of Bioinformatics, Division of Cancer Genomics, National Cancer Center Research Institute, Tokyo, Japan; 441 A.A. Kharkevich Institute of Information Transmission Problems, Moscow, Russia; 442 Oncology and Immunology, Dmitry Rogachev National Research Center of Pediatric Hematology, Moscow, Russia; 443 Skolkovo Institute of Science and Technology, Moscow, Russia; 444 Department of Surgery, The George Washington University, School of Medicine and Health Science, Washington, DC 20052 USA; 445 Endocrine Oncology Branch, Center for Cancer Research, National Cancer Institute, National Institutes of Health, Bethesda, MD 20892 USA; 446 Melanoma Institute Australia, Macquarie University, Sydney, NSW 2109 Australia; 447 MIT Computer Science and Artificial Intelligence Laboratory, Massachusetts Institute of Technology, Cambridge, MA 02139 USA; 448 Tissue Pathology and Diagnostic Oncology, Royal Prince Alfred Hospital, Sydney, NSW 2050 Australia; 449 Cholangiocarcinoma Screening and Care Program and Liver Fluke and Cholangiocarcinoma Research Centre, Faculty of Medicine, Khon Kaen University, Khon Kaen, 40002 Thailand; 450 Controlled Department and Institution, New York, NY USA; 451 Englander Institute for Precision Medicine, Weill Cornell Medicine, New York, NY 10065 USA; 452 National Cancer Center, Gyeonggi, South Korea; 453 Health Sciences Department of Biomedical Informatics, University of California San Diego, La Jolla, CA 92093 USA; 454 Research Core Center, National Cancer Centre Korea, Goyang-si, South Korea; 455 Department of Health Sciences and Technology, Sungkyunkwan University School of Medicine, Seoul, South Korea; 456 Samsung Genome Institute, Seoul, South Korea; 457 Breast Oncology Program, Dana-Farber/Brigham and Women’s Cancer Center, Boston, MA 02215 USA; 458 Department of Surgery, Memorial Sloan Kettering Cancer Center, New York, NY 10065 USA; 459 Division of Breast Surgery, Brigham and Women’s Hospital, Boston, MA 02115 USA; 460 Integrative Bioinformatics Support Group, National Institute of Environmental Health Sciences (NIEHS), Durham, NC 27709 USA; 461 Department of Clinical Science, University of Bergen, 5007 Bergen, Norway; 462 Center For Medical Innovation, Seoul National University Hospital, Seoul, South Korea; 463 Department of Internal Medicine, Seoul National University Hospital, Seoul, South Korea; 464 Institute of Computer Science, Polish Academy of Sciences, Warsawa, Poland; 465 Functional and Structural Genomics, German Cancer Research Center (DKFZ), 69120 Heidelberg, Germany; 466 Laboratory of Translational Genomics, Division of Cancer Epidemiology and Genetics, National Cancer Institute, National Institutes of Health, Bethesda, MD 20892 USA; 467 Institute for Medical Informatics Statistics and Epidemiology, University of Leipzig, 04109 Leipzig, Germany; 468 Morgan Welch Inflammatory Breast Cancer Research Program and Clinic, The University of Texas MD Anderson Cancer Center, Houston, TX 77030 USA; 469 Department of Hematology and Oncology, Georg-Augusts-University of Göttingen, 37073 Göttingen, Germany; 470 Institute of Cell Biology (Cancer Research), University of Duisburg-Essen, 47057 Essen, Germany; 471 King’s College London and Guy’s and St. Thomas’ NHS Foundation Trust, London, WC2R 2LS UK; 472 Center for Epigenetics, Van Andel Research Institute, Grand Rapids, MI 49503 USA; 473 The University of Queensland Centre for Clinical Research, Royal Brisbane and Women’s Hospital, Herston, QLD 4029 Australia; 474 Department of Pediatric Oncology and Hematology, University of Cologne, 50923 Cologne, Germany; 475 University of Düsseldorf, 40225 Düsseldorf, Germany; 476 Department of Pathology, Institut Jules Bordet, Brussels, 1000 Belgium; 477 Institute of Biomedicine, Sahlgrenska Academy at University of Gothenburg, 413 90 Gothenburg, Sweden; 478 Children’s Medical Research Institute, Sydney, NSW 2145 Australia; 479 ILSbio, LLC Biobank, Chestertown, MD USA; 480 Division of Genetics and Genomics, Boston Children’s Hospital, Harvard Medical School, Boston, MA 02115 USA; 481 Institute for Bioengineering and Biopharmaceutical Research (IBBR), Hanyang University, Seoul, South Korea; 482 Department of Statistics, University of California Santa Cruz, Santa Cruz, CA 95064 USA; 483 Department of Vertebrate Genomics/Otto Warburg Laboratory Gene Regulation and Systems Biology of Cancer, Max Planck Institute for Molecular Genetics, 14195 Berlin, Germany; 484 McGill University and Genome Quebec Innovation Centre, Montreal, QC H3A 0G4 Canada; 485 Gynecologic Oncology, NYU Laura and Isaac Perlmutter Cancer Center, New York University, New York, NY 10003 USA; 486 Division of Oncology, Stem Cell Biology Section, Washington University School of Medicine, St. Louis, MO 63110 USA; 487 Harvard University, Cambridge, MA 02138 USA; 488 Urologic Oncology Branch, Center for Cancer Research, National Cancer Institute, National Institutes of Health, Bethesda, MD 20892 USA; 489 University of Oslo, 0315 Oslo, Norway; 490 University of Toronto, Toronto, ON M5S Canada; 491 School of Life Sciences, Peking University, Beijing, China; 492 Leidos Biomedical Research, Inc, McLean, VA USA; 493 Hematology, Hospital Clinic, Institut d’Investigacions Biomèdiques August Pi i Sunyer (IDIBAPS), University of Barcelona, 08007 Barcelona, Spain; 494 Second Military Medical University, Shanghai, China; 495 Chinese Cancer Genome Consortium, Shenzhen, China; 496 Department of Medical Oncology, Beijing Hospital, Beijing, China; 497 Laboratory of Molecular Oncology, Key Laboratory of Carcinogenesis and Translational Research (Ministry of Education), Peking University Cancer Hospital and Institute, Beijing, China; 498 School of Medicine/School of Mathematics and Statistics, University of St. Andrews, St. Andrews, Fife KY16 9AJ UK; 499 Department of Biochemistry and Molecular Biology, Faculty of Medicine, University Institute of Oncology-IUOPA, Oviedo, Spain; 500 Institut Bergonié, Bordeaux, France; 501 Cancer Unit, MRC University of Cambridge, Cambridge, CB2 1TN UK; 502 Department of Pathology and Laboratory Medicine, Center for Personalized Medicine, Children’s Hospital Los Angeles, Los Angeles, CA 90027 USA; 503 John Curtin School of Medical Research, Canberra, ACT 2601 Australia; 504 MVZ Department of Oncology, PraxisClinic am Johannisplatz, Leipzig, Germany; 505 Department of Information Technology, Ghent University, Ghent, Belgium; 506 Department of Plant Biotechnology and Bioinformatics, Ghent University, Ghent, Belgium; 507 Institute for Genomic Medicine, Nationwide Children’s Hospital, Columbus, OH 43205 USA; 508 Department of Surgery, Duke University, Durham, NC 27708 USA; 509 Institut Català de Paleontologia Miquel Crusafont, Universitat Autònoma de Barcelona, Barcelona, Spain; 510 University of Glasgow, Glasgow, G12 8QQ UK; 511 Institut d’Investigacions Biomèdiques August Pi i Sunyer (IDIBAPS), Barcelona, Spain; 512 Department of Surgery and Cancer, Imperial College, London, INY SW7 2BU UK; 513 Applications Department, Oxford Nanopore Technologies, Oxford, OX4 4DQ UK; 514 Department of Obstetrics, Gynecology and Reproductive Services, University of California San Francisco, San Francisco, CA 94110 USA; 515 Department of Biochemistry and Molecular Medicine, University California at Davis, Sacramento, CA 95616 USA; 516 STTARR Innovation Facility, Princess Margaret Cancer Centre, Toronto, ON M5G 2C1 Canada; 517 Discipline of Surgery, Western Sydney University, Penrith, NSW 2150 Australia; 518 Department of Genetics, Lineberger Comprehensive Cancer Center, University of North Carolina at Chapel Hill, Chapel Hill, NC 27599 USA; 519 Departments of Neurology and Neurosurgery, Henry Ford Hospital, Detroit, MI 48202 USA; 520 Institute of Pathology, University Medical Center Hamburg-Eppendorf, 20251 Hamburg, Germany; 521 Department of Health Sciences, Faculty of Medical Sciences, Kyushu University, Fukuoka, Japan; 522 Heidelberg Academy of Sciences and Humanities, Heidelberg, Germany; 523 Department of Clinical Pathology, University of Melbourne, Melbourne, VIC 3010 Australia; 524 Department of Pathology, Roswell Park Cancer Institute, Buffalo, NY 14203 USA; 525 Department of Computer Science, University of Helsinki, 00100 Helsinki, Finland; 526 Institute of Biotechnology, University of Helsinki, 00100 Helsinki, Finland; 527 Organismal and Evolutionary Biology Research Programme, University of Helsinki, 00100 Helsinki, Finland; 528 Department of Obstetrics and Gynecology, Division of Gynecologic Oncology, Washington University School of Medicine, St. Louis, MO 63110 USA; 529 Penrose St. Francis Health Services, Colorado Springs, CO 80923 USA; 530 Institute of Pathology, Ulm University and University Hospital of Ulm, Ulm, 89081 Germany; 531 National Cancer Center, Tokyo, Japan; 532 Genome Institute of Singapore, Singapore, 138672 Singapore; 533 German Cancer Aid, Bonn, Germany; 534 Programme in Cancer and Stem Cell Biology, Centre for Computational Biology, Duke-NUS Medical School, 169857 Singapore, Singapore; 535 The Chinese University of Hong Kong, Shatin, NT, Hong Kong, China; 536 Fourth Military Medical University, Shaanxi, China; 537 The University of Cambridge School of Clinical Medicine, Cambridge, CB2 1TN UK; 538 St. Jude Children’s Research Hospital, Memphis, TN 38105 USA; 539 University Health Network, Princess Margaret Cancer Centre, Toronto, ON M5G 1L7 Canada; 540 Center for Biomolecular Science and Engineering, University of California Santa Cruz, Santa Cruz, CA 95064 USA; 541 Department of Medicine, University of Chicago, Chicago, IL 60637 USA; 542 Department of Neurology, Mayo Clinic, Rochester, MN 55905 USA; 543 Cambridge Oesophagogastric Centre, Cambridge University Hospitals NHS Foundation Trust, Cambridge, CB2 0QQ UK; 544 Department of Computer Science, Carleton College, Northfield, MN 55057 USA; 545 Institute of Cancer Sciences, College of Medical Veterinary and Life Sciences, University of Glasgow, Glasgow, G12 8QQ UK; 546 Department of Epidemiology, University of Alabama at Birmingham, Birmingham, AL 35294 USA; 547 HudsonAlpha Institute for Biotechnology, Huntsville, AL 35806 USA; 548 O’Neal Comprehensive Cancer Center, University of Alabama at Birmingham, Birmingham, AL 35294 USA; 549 Department of Pathology, Keio University School of Medicine, Tokyo, Japan; 550 Department of Hepatobiliary and Pancreatic Oncology, National Cancer Center Hospital, Tokyo, Japan; 551 Sage Bionetworks, Seattle, WA USA; 552 Lymphoma Genomic Translational Research Laboratory, National Cancer Centre, Singapore, 98121 Singapore; 553 Department of Clinical Pathology, Robert-Bosch-Hospital, 70376 Stuttgart, Germany; 554 Department of Cell and Systems Biology, University of Toronto, Toronto, ON M5S Canada; 555 Department of Biosciences and Nutrition, Karolinska Institutet, 171 77 Stockholm, Sweden; 556 Center for Liver Cancer, Research Institute and Hospital, National Cancer Center, Gyeonggi, South Korea; 557 Division of Hematology-Oncology, Samsung Medical Center, Sungkyunkwan University School of Medicine, Seoul, South Korea; 558 Samsung Advanced Institute for Health Sciences and Technology, Sungkyunkwan University School of Medicine, Seoul, South Korea; 559 Cheonan Industry-Academic Collaboration Foundation, Sangmyung University, Cheonan, South Korea; 560 NYU Langone Medical Center, New York, NY 10016 USA; 561 Department of Hematology and Medical Oncology, Cleveland Clinic, Cleveland, OH 44195 USA; 562 Department of Health Sciences Research, Mayo Clinic, Rochester, MN 55905 USA; 563 Helen F. Graham Cancer Center at Christiana Care Health Systems, Newark, DE 19713 USA; 564 Heidelberg University Hospital, 69120 Heidelberg, Germany; 565 CSRA Incorporated, Fairfax, VA USA; 566 Research Department of Pathology, University College London Cancer Institute, London, WC1E 6DD UK; 567 Department of Research Oncology, Guy’s Hospital, King’s Health Partners AHSC, King’s College London School of Medicine, London, WC2R 2LS UK; 568 Faculty of Medicine and Health Sciences, Macquarie University, Sydney, NSW 2109 Australia; 569 University Hospital of Minjoz, INSERM UMR 1098, Besançon, France; 570 Spanish National Cancer Research Centre, Madrid, 28029 Spain; 571 Center of Digestive Diseases and Liver Transplantation, Fundeni Clinical Institute, Bucharest, Romania; 572 Cureline, Inc, South San Francisco, CA USA; 573 St. Luke’s Cancer Centre, Royal Surrey County Hospital NHS Foundation Trust, Guildford, UK; 574 Cambridge Breast Unit, Addenbrooke’s Hospital, Cambridge University Hospital NHS Foundation Trust and NIHR Cambridge Biomedical Research Centre, Cambridge, CB2 0QQ UK; 575 East of Scotland Breast Service, Ninewells Hospital, Aberdeen, UK; 576 Department of Genetics, Microbiology and Statistics, University of Barcelona, IRSJD, IBUB, Barcelona, Spain; 577 Department of Obstetrics and Gynecology, Medical College of Wisconsin, Milwaukee, WI 53226 USA; 578 Hematology and Medical Oncology, Winship Cancer Institute of Emory University, Atlanta, GA 30322 USA; 579 Department of Computer Science, Princeton University, Princeton, NJ 08544 USA; 580 Vanderbilt Ingram Cancer Center, Vanderbilt University, Nashville, TN 37235 USA; 581 Ohio State University College of Medicine and Arthur G. James Comprehensive Cancer Center, Columbus, OH 43210 USA; 582 Department of Surgery, Yokohama City University Graduate School of Medicine, Kanagawa, 236-0027 Japan; 583 Research Computing Center, University of North Carolina at Chapel Hill, Chapel Hill, NC 27599 USA; 584 School of Molecular Biosciences and Center for Reproductive Biology, Washington State University, Pullman, WA 99163 USA; 585 Department of Laboratory Medicine and Pathobiology, University of Toronto, Toronto, ON M5S Canada; 586 Department of Pathology, Human Oncology and Pathogenesis Program, Memorial Sloan Kettering Cancer Center, New York, NY 10065 USA; 587 University Hospital Giessen, Pediatric Hematology and Oncology, Giessen, Germany; 588 Oncologie Sénologie, ICM Institut Régional du Cancer, Montpellier, France; 589 Institute of Clinical Molecular Biology, Christian-Albrechts-University, Kiel, Germany; 590 Institute of Pathology, University of Wuerzburg, 97070 Wuerzburg, Germany; 591 Department of Urology, North Bristol NHS Trust, Bristol, UK; 592 SingHealth, Duke-NUS Institute of Precision Medicine, National Heart Centre Singapore, Singapore, 169609 Singapore; 593 Bern Center for Precision Medicine, University Hospital of Bern, University of Bern, Bern, 3012 Switzerland; 594 Englander Institute for Precision Medicine, Weill Cornell Medicine and New York Presbyterian Hospital, New York, NY 10065 USA; 595 Meyer Cancer Center, Weill Cornell Medicine, New York, NY 10065 USA; 596 Pathology and Laboratory, Weill Cornell Medical College, New York, NY 10065 USA; 597 Vall d’Hebron Institute of Oncology: VHIO, Barcelona, Spain; 598 General and Hepatobiliary-Biliary Surgery, Pancreas Institute, University and Hospital Trust of Verona, Verona, Italy; 599 National Centre for Biological Sciences, Tata Institute of Fundamental Research, Bangalore, 560065 India; 600 Department of Pathology, GZA-ZNA Hospitals, Antwerp, Belgium; 601 Analytical Biological Services, Inc, Wilmington, DE 19801 USA; 602 Sydney Medical School, University of Sydney, Sydney, NSW 2050 Australia; 603 cBio Center, Dana-Farber Cancer Institute, Harvard Medical School, Boston, MA 02115 USA; 604 Department of Cell Biology, Harvard Medical School, Boston, MA 02115 USA; 605 Advanced Centre for Treatment Research and Education in Cancer, Tata Memorial Centre, Navi Mumbai, Maharashtra 400012 India; 606 School of Environmental and Life Sciences, Faculty of Science, The University of Newcastle, Ourimbah, NSW 2308 Australia; 607 Department of Dermatology, University Hospital of Essen, 45147 Essen, Germany; 608 Martini-Clinic, Prostate Cancer Center, University Medical Center Hamburg-Eppendorf, 20251 Hamburg, Germany; 609 Department of General Internal Medicine, University of Kiel, 24118 Kiel, Germany; 610 German Cancer Consortium (DKTK), Partner site Berlin, Berlin, Germany; 611 Cancer Research Institute, Beth Israel Deaconess Medical Center, Boston, MA 02215 USA; 612 University of Pittsburgh, Pittsburgh, PA 15260 USA; 613 Department of Ophthalmology and Ocular Genomics Institute, Massachusetts Eye and Ear, Harvard Medical School, Boston, MA 02115 USA; 614 Center for Psychiatric Genetics, NorthShore University HealthSystem, Evanston, IL 60031 USA; 615 Van Andel Research Institute, Grand Rapids, MI 49503 USA; 616 Laboratory of Molecular Medicine, Human Genome Center, Institute of Medical Science, University of Tokyo, Tokyo, Japan; 617 Japan Agency for Medical Research and Development, Tokyo, Japan; 618 Korea University, Seoul, South Korea; 619 Murtha Cancer Center, Walter Reed National Military Medical Center, Bethesda, MD 20814 USA; 620 Human Genetics, University of Kiel, 24118 Kiel, Germany; 621 Department of Oncologic Pathology, Dana-Farber Cancer Institute, Harvard Medical School, Boston, MA 02115 USA; 622 Oregon Health and Science University, Portland, OR 97239 USA; 623 Center for RNA Interference and Noncoding RNA, The University of Texas MD Anderson Cancer Center, Houston, TX 77030 USA; 624 Department of Experimental Therapeutics, The University of Texas MD Anderson Cancer Center, Houston, TX 77030 USA; 625 Department of Gynecologic Oncology and Reproductive Medicine, The University of Texas MD Anderson Cancer Center, Houston, TX 77030 USA; 626 University Hospitals Coventry and Warwickshire NHS Trust, Coventry, UK; 627 Department of Radiation Oncology, Radboud University Nijmegen Medical Centre, Nijmegen, 6525 GA The Netherlands; 628 Institute for Genomics and Systems Biology, University of Chicago, Chicago, IL USA; 629 Clinic for Hematology and Oncology, St.-Antonius-Hospital, 60637 Eschweiler, Germany; 630 Computational and Systems Biology Program, Memorial Sloan Kettering Cancer Center, New York, NY 10065 USA; 631 University of Iceland, Reykjavik, Iceland; 632 Division of Computational Genomics and Systems Genetics, German Cancer Research Center (DKFZ), Heidelberg, Germany; 633 Dundee Cancer Centre, Ninewells Hospital, Dundee, UK; 634 Department for Internal Medicine III, University of Ulm and University Hospital of Ulm, Ulm, Germany; 635 Institut Curie, INSERM Unit 830, Paris, France; 636 Department of Gastroenterology and Hepatology, Yokohama City University Graduate School of Medicine, Kanagawa, Japan; 637 Department of Laboratory Medicine, Radboud University Nijmegen Medical Centre, Nijmegen, 6525 GA The Netherlands; 638 Division of Cancer Genome Research, German Cancer Research Center (DKFZ), Heidelberg, Germany; 639 Department of General Surgery, Singapore General Hospital, Singapore, Singapore; 640 Cancer Science Institute of Singapore, National University of Singapore, Singapore, Singapore; 641 Department of Medical and Clinical Genetics, Genome-Scale Biology Research Program, University of Helsinki, Helsinki, Finland; 642 East Anglian Medical Genetics Service, Cambridge University Hospitals NHS Foundation Trust, Cambridge, UK; 643 Irving Institute for Cancer Dynamics, Columbia University, New York, NY 10027 USA; 644 Institute of Molecular and Cell Biology, Singapore, Singapore; 645 Laboratory of Cancer Epigenome, Division of Medical Science, National Cancer Centre Singapore, Singapore, Singapore; 646 Universite Lyon, INCa-Synergie, Centre Léon Bérard, Lyon, France; 647 Department of Urology, Mayo Clinic, Rochester, MN 55905 USA; 648 Royal National Orthopaedic Hospital-Stanmore, Stanmore, Middlesex UK; 649 Giovanni Paolo II / I.R.C.C.S. Cancer Institute, Bari, BA Italy; 650 Neuroblastoma Genomics, German Cancer Research Center (DKFZ), Heidelberg, Germany; 651 Fondazione Policlinico Universitario Gemelli IRCCS, Rome, Italy, Rome, Italy; 652 University of Verona, Verona, Italy; 653 Centre National de Génotypage, CEA-Institute de Génomique, Evry, France; 654 CAPHRI Research School, Maastricht University, Maastricht, ER The Netherlands; 655 Department of Biopathology, Centre Léon Bérard, Lyon, France; 656 Université Claude Bernard Lyon 1, Villeurbanne, France; 657 Core Research for Evolutional Science and Technology (CREST), JST, Tokyo, Japan; 658 Department of Biological Sciences, Laboratory for Medical Science Mathematics, Graduate School of Science, University of Tokyo, Yokohama, Japan; 659 Department of Medical Science Mathematics, Medical Research Institute, Tokyo Medical and Dental University (TMDU), Tokyo, Japan; 660 University Hospitals Birmingham NHS Foundation Trust, Birmingham, UK; 661 Centre for Cancer Research and Cell Biology, Queen’s University, Belfast, UK; 662 Breast Medical Oncology, The University of Texas MD Anderson Cancer Center, Houston, TX 77030 USA; 663 Department of Surgery, Johns Hopkins University School of Medicine, Baltimore, MD 21205 USA; 664 Department of Oncology-Pathology, Science for Life Laboratory, Karolinska Institute, 171 77 Stockholm, Sweden; 665 School of Cancer Sciences, Faculty of Medicine, University of Southampton, Southampton, UK; 666 Department of Gene Technology, Tallinn University of Technology, Tallinn, Estonia; 667 Genetics and Genome Biology Program, SickKids Research Institute, The Hospital for Sick Children, Toronto, ON M5G 1X8 Canada; 668 Departments of Neurosurgery and Hematology and Medical Oncology, Winship Cancer Institute and School of Medicine, Emory University, Atlanta, GA 30322 USA; 669 Department of Clinical and Molecular Medicine, Faculty of Medicine and Health Sciences, Norwegian University of Science and Technology, Trondheim, Norway; 670 Argmix Consulting, North Vancouver, BC Canada; 671 Department of Information Technology, Ghent University, Interuniversitair Micro-Electronica Centrum (IMEC), Ghent, Belgium; 672 Nuffield Department of Surgical Sciences, John Radcliffe Hospital, University of Oxford, Oxford, OX1 2JD UK; 673 Institute of Mathematics and Computer Science, University of Latvia, Riga, LV 1586 Latvia; 674 Discipline of Pathology, Sydney Medical School, University of Sydney, Sydney, NSW 2006 Australia; 675 Department of Applied Mathematics and Theoretical Physics, Centre for Mathematical Sciences, University of Cambridge, Cambridge, 10027 UK; 676 Department of Statistics, Columbia University, New York, NY USA; 677 Department of Immunology, Genetics and Pathology, Science for Life Laboratory, Uppsala University, 752 36 Uppsala, Sweden; 678 Department of Histopathology, Cambridge University Hospitals NHS Foundation Trust, Cambridge, CB2 0QQ UK; 679 Oxford NIHR Biomedical Research Centre, University of Oxford, Oxford, OX1 2JD UK; 680 Georgia Regents University Cancer Center, Augusta, GA 30912 USA; 681 Wythenshawe Hospital, Manchester, M23 9LT UK; 682 Wellcome Centre for Human Genetics, University of Oxford, Oxford, OX1 2JD UK; 683 Thoracic Oncology Laboratory, Mayo Clinic, Rochester, MN 55905 USA; 684 Institute for Genomic Medicine, Nationwide Children’s Hospital, Columbus, OH 43205 USA; 685 Department of Obstetrics and Gynecology, Division of Gynecologic Oncology, Mayo Clinic, Rochester, MN 55905 USA; 686 International Institute for Molecular Oncology, Poznań, 60-203 Poland; 687 Poznan University of Medical Sciences, 61-701 Poznań, Poland; 688 Genomics and Proteomics Core Facility High Throughput Sequencing Unit, German Cancer Research Center (DKFZ), 69120 Heidelberg, Germany; 689 NCCS-VARI Translational Research Laboratory, National Cancer Centre Singapore, Singapore, 169610 Singapore; 690 Edison Family Center for Genome Sciences and Systems Biology, Washington University, St. Louis, MO 63130 USA; 691 MRC-University of Glasgow Centre for Virus Research, Glasgow, G61 1QH UK; 692 Department of Medical Informatics and Clinical Epidemiology, Division of Bioinformatics and Computational Biology, OHSU Knight Cancer Institute, Oregon Health and Science University, Portland, OR 97239 USA; 693 School of Electronic Information and Communications, Huazhong University of Science and Technology, 430074 Wuhan, China; 694 Department of Applied Mathematics and Statistics, Johns Hopkins University, Baltimore, MD 21218 USA; 695 Department of Cancer Genome Informatics, Graduate School of Medicine, Osaka University, Osaka, 565-0871 Japan; 696 School of Mathematics and Statistics, University of Sydney, Sydney, NSW 2006 Australia; 697 Ben May Department for Cancer Research and Department of Human Genetics, University of Chicago, Chicago, IL 60637 USA; 698 Tri-Institutional PhD Program in Computational Biology and Medicine, Weill Cornell Medicine, New York, NY 10065 USA; 699 Department of Medicine and Therapeutics, The Chinese University of Hong Kong, Shatin, NT, Hong Kong, China; 700 Department of Biostatistics, The University of Texas MD Anderson Cancer Center, Houston, TX 77030 USA; 701 Duke-NUS Medical School, Singapore, 169857 Singapore; 702 Department of Surgery, Ruijin Hospital, Shanghai Jiaotong University School of Medicine, Shanghai, China; 703 School of Computing Science, University of Glasgow, Glasgow, G12 8QQ UK; 704 Division of Orthopaedic Surgery, Oslo University Hospital, 0450 Oslo, Norway; 705 Eastern Clinical School, Monash University, Melbourne, VIC 3800 Australia; 706 Epworth HealthCare, Richmond, VIC 3121 Australia; 707 Department of Biostatistics and Computational Biology, Dana-Farber Cancer Institute and Harvard Medical School, Boston, MA 02215 USA; 708 Department of Biomedical Informatics, College of Medicine, The Ohio State University, Columbus, OH 43202 USA; 709 The Ohio State University Comprehensive Cancer Center (OSUCCC–James), Columbus, OH 43202 USA; 710 BIOPIC, ICG and College of Life Sciences, Peking University, 100871 Beijing, China; 711 The University of Texas School of Biomedical Informatics (SBMI) at Houston, Houston, TX 77030 USA; 712 Department of Biostatistics, University of North Carolina at Chapel Hill, Chapel Hill, NC 27599 USA; 713 Department of Biochemistry and Molecular Genetics, Feinberg School of Medicine, Northwestern University, Chicago, IL 60208 USA; 714 Faculty of Medicine and Health, University of Sydney, Sydney, NSW 2006 Australia; 715 Department of Pathology, Erasmus Medical Center Rotterdam, 1066 CX Rotterdam, GD The Netherlands; 716 Division of Molecular Carcinogenesis, The Netherlands Cancer Institute, 1066 CX Amsterdam, The Netherlands; 717 Institute of Molecular Life Sciences and Swiss Institute of Bioinformatics, University of Zurich, 8006 Zurich, Switzerland

**Keywords:** Comparative genomics, Communication and replication, Cancer genomics, Genetic databases

## Abstract

The Cancer Genome Atlas (TCGA) and International Cancer Genome Consortium (ICGC) curated consensus somatic mutation calls using whole exome sequencing (WES) and whole genome sequencing (WGS), respectively. Here, as part of the ICGC/TCGA Pan-Cancer Analysis of Whole Genomes (PCAWG) Consortium, which aggregated whole genome sequencing data from 2,658 cancers across 38 tumour types, we compare WES and WGS side-by-side from 746 TCGA samples, finding that ~80% of mutations overlap in covered exonic regions. We estimate that low variant allele fraction (VAF < 15%) and clonal heterogeneity contribute up to 68% of private WGS mutations and 71% of private WES mutations. We observe that ~30% of private WGS mutations trace to mutations identified by a single variant caller in WES consensus efforts. WGS captures both ~50% more variation in exonic regions and un-observed mutations in loci with variable GC-content. Together, our analysis highlights technological divergences between two reproducible somatic variant detection efforts.

## Abstract (web summary)

With the generation of large pan-cancer whole-exome and whole-genome sequencing projects, a question remains about how comparable these datasets are. Here, using The Cancer Genome Atlas samples analysed as part of the Pan-Cancer Analysis of Whole Genomes project, the authors explore the concordance of mutations called by whole exome sequencing and whole genome sequencing techniques.

## Introduction

Complementary efforts of The Cancer Genome Atlas (TCGA) and the International Cancer Genome Consortium (ICGC) have recently produced two of the highest quality and most elaborate and reproducible somatic variant call sets from exome and whole genome-level data in cancer genomics, respectively. The motivation for these efforts stems from the notion that “scientific crowd sourcing” and combining mutation callers can provide very strong results.

These two efforts produced variant calls from 10 different callers, namely Radia^1^, Varscan^2^, MuSE^3^, MuTect^4^, Pindel^5,6^, Indelocator^7^, SomaticSniper^8^ for WES and MuSE, Broad-Pipeline (anchored by MuTect), Sanger-pipeline, German Cancer Research Center pipeline (DKFZ), and SMuFin^9^, for WGS. Briefly, the PCAWG Consortium aggregated whole genome sequencing data from 2658 cancers across 38 tumor types generated by the ICGC and TCGA projects. These sequencing data were re-analyzed with standardized, high-accuracy pipelines to align to the human genome (reference build hs37d5) and identify germline variants and somatically acquired mutations^10^. Of the 885 TCGA samples in ICGC, 746 were included in the latest exome call set produced by both the Multi-Center Mutation Calling in Multiple Cancers (MC3) effort and the Pan-Cancer Analysis of Whole Genomes (PCAWG) Consortium set. These 746 samples represent a critical benchmark for high-level analysis of similarities and differences between exome and genome somatic variant detection methods.

Reproducibility of mutations identified by both whole exome capture sequencing and whole genome sequencing (WGS) techniques remains an important issue, not only for the scientific use of large, established data sets, but for data designs of future research projects. Previous work analyzing exome capture effects on sequence read quality has shown that GC-content bias is the major source of variation in coverage^11^. A performance comparison across exome-captured platforms demonstrated that for most technologies, both high and low GC-content result in reduced coverage in read depth^12^. Belkadi et al. compared mutation calls between WGS and WES, observing that ~3% of coding variants with high quality were only detected in WGS, and WGS also had a more uniform distribution of coverage depth, genotype quality, and minor read ratio^13^. Furthermore, due to the relatively high error rate per read in next-generation sequencing^14^, the detectability of mutations with low variant allele fractions (VAFs) is limited by background noise. Despite these studies’ nuanced preference towards WGS, others contend that WES will remain a better choice until costs of WGS fall^15^. The decision to sequence exomes or whole genomes is further confounded as more recent publications in oncology select either WGS^16–20^ or WES^21–24^. Recognizing the unresolved nature of this issue, Schwarze et al. have called for more comprehensive studies comparing the WES and WGS studies, especially as this issue has important ramifications for the clinic^25^.

Our analysis provides confidence that mutation calls within the captured exonic regions of these two data sets are largely consistent. We highlight common sample, cohort, and caller-specific challenges in cancer variant detection from the TCGA and ICGC efforts. We show that variants that are most confidently called in one database i.e., called by multiple callers, are very likely to be called in the other. We assess how reproducibility impacts higher-level mutation signature analysis and illustrate the need for caution in assessing performance that can only be identified by the overlap of these two data sets. Finally, we explore the capacity of WGS to detect recurrent non-coding mutations captured by whole exome sequencing.

## Results

### Data and workflow

**Fig. 1:**
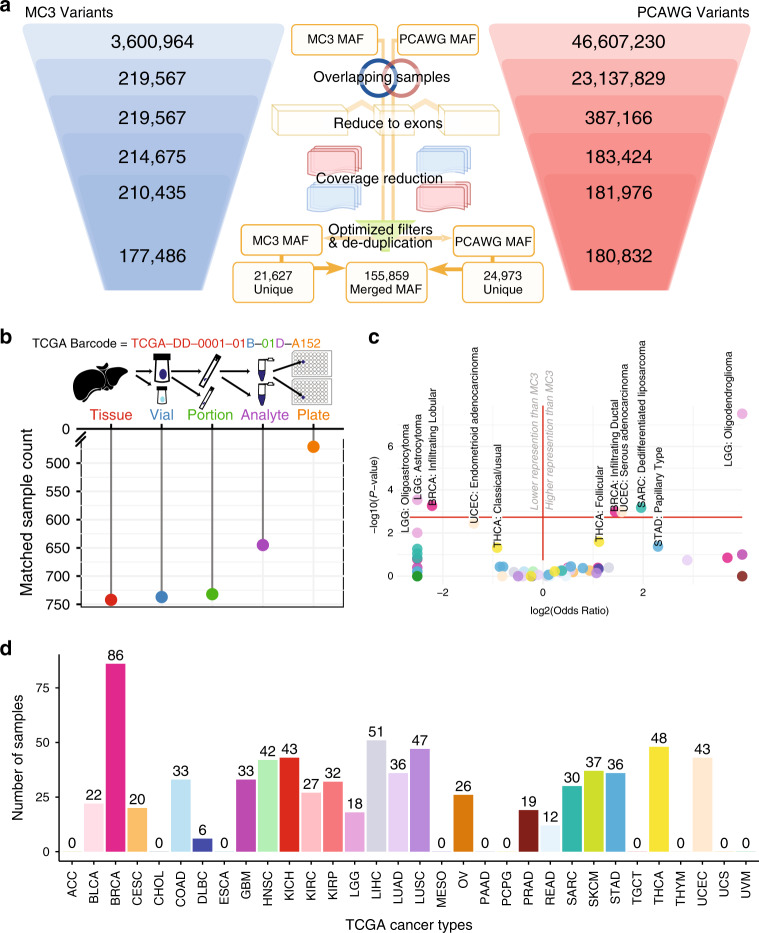
Workflow and sample inclusion statistics. **a** A workflow diagram illustrates the number of mutations present during each step (gradient) of the filtering processes for MC3 (left, blue) and PCAWG (right, red). A brief description of each step of the intersection process is shown in between. **b** TCGA barcodes and aliquot IDs were used to match somatic sequencing. The exact match of these IDs is shown for various collection aliquots from tissue to plate. **c** A volcano plot highlights cancer subtype discrepancy between each PCAWG and MC3 with −log10(*p*-value) on the *y*-axis and log2(odds ratio) on the *x*-axis (Fisher’s exact test). The horizontal red bar indicates a significant threshold after multiple testing correction. Positive values indicate an over-representation of a cancer subtype in PCAWG, while negative values indicate an under-representation of a cancer subtype in PCAWG compared to MC3-separated by a vertical red bar. **d** Sample counts for each cancer type are shown in a bar chart. The colors coordinate with panel **c**.

We used publicly available data from the MC3 and PCAWG repositories, consisting of ~3.6 M and ~47 M variants, respectively (Fig. 1a). 746 samples were sequenced by both WES and WGS, comprising various aliquots and portions of the same tumor (Supplementary Data 1, Fig. 1b). Effects of these differences are discussed below for preliminary results, but we ultimately used the entire set of 746 samples in the variant overlap analysis, since the effects of tumor partitioning did not play a significant role (Supplementary Fig. 1). By restricting the public data sets to overlapping samples, we reduced the total corpus to ~220 K (6.1%) and ~23 M (49.6%) mutations for exome and whole genome, respectively. It is notable that there is an enrichment of variants in hypermutated samples from COAD, HNSC, LUAD, and STAD in the PCAWG set used in this study (Supplementary Fig. 2). To begin building a comparable set of mutations between these two studies, we further restricted the whole genome data set to exon regions provided by the MC3 analysis working group. This reduced the WGS data set to 1.6% of its original size, within range of total exome material estimations^26^ (Fig. 1a). The next step involved removing poorly-covered variants potentially caused by technical anomalies by limiting mutations to those captured in coverage files (distributed as.wig files). A reciprocal coverage strategy was used, meaning PCAWG mutations were restricted to covered genomic regions in MC3 and vice versa, thereby maintaining a complementary set of callable genomic regions. Removal of mutations in uncovered regions reduced the remaining PCAWG data set by approximately one-half, from 387,166 to 183,424 mutations. We also identified 4241 MC3 and 2219 PCAWG mutations that were present in the respective MAF but were not marked as covered in the coverage files provided by a single group. This suggests that different tools consider different minimum coverage strategies. These mutations reflect 2.0% and 1.2%, respectively, of the total mutation discrepancy and were removed because some callers had limited capacity to identify mutations in poorly-covered regions (see “Methods” section). Finally, filter flags provided by MC3 were used to assess somatic mutation filtering strategies. At this stage, we performed filter optimization to comprehensively evaluate all possible combinations of MC3 filters (Supplementary Fig. 3a). Ultimately, we decided to only remove OxoG labeled artifacts and duplicated events produced by these filters (see “Methods” section, Supplementary Data 2). Since each stage of this filtering workflow resulted in many alternative decisions and outcomes, we built MAFit, a web-based graphical user interface that allows users to easily customize comparisons of merged mutations (https://mbailey.shinyapps.io/MAFit/). A MC3 filter assessment also shows that many variants with filter flags in MC3 are present in the PCAWG variant call set, suggesting a need for improved filtering strategies (Supplementary Fig. 3b).

TCGA samples comprise a sizable fraction of the PCAWG sample pool (~30%, Supplementary Data 1) Additional WGS sequencing from TCGA allowed for mutation validation^27^ and insights into non-coding mutations, such as in *TET2*. However, this selection process could have potentially influenced our basic comparison of exome-sequenced samples and genome-sequenced samples in two fundamental ways. First, vagaries of tumor extraction and tissue storage protocols may have resulted in many different portions of a tumor being stored, introducing the possibility that different subclones of the same tumor could be present. These could have very different genetic makeups. This information was captured in different substrings of the TCGA identification barcode (see “Methods” section). From the 746 TCGA barcodes, we found that 64% (477) could be traced to the same well of a microtiter plate (Fig. 1b). After correcting for cancer type, we modeled both the impact of matching barcode identifiers between MC3 and PCAWG and variant concordance, finding that differing barcodes did not have an appreciable impact. This result was seen for all samples, even when excluding the hypermutator (Fig. 1). Second, each AWG was able to independently select samples for WGS, which, while not affecting mutation calling, does raise potential biases when comparing PCAWG results to TCGA exome cohort data. An enrichment analysis was performed to identify which tumor subtypes may have been preferentially selected for different cancer types. We found that four tumor subtypes were enriched in the PCAWG effort from TCGA samples: infiltrating ductal breast cancer, endometrial serous adenocarcinoma, differentiated liposarcoma, and low grade oligodendroglioma (FDR < 0.05, Fig. 1c, Supplementary Data 3, and see “Methods” section). Final tumor sample counts for each cancer type are shown in Fig. 1d.

### Landscape of mutational overlap between WGS and WES calls

**Fig. 2:**
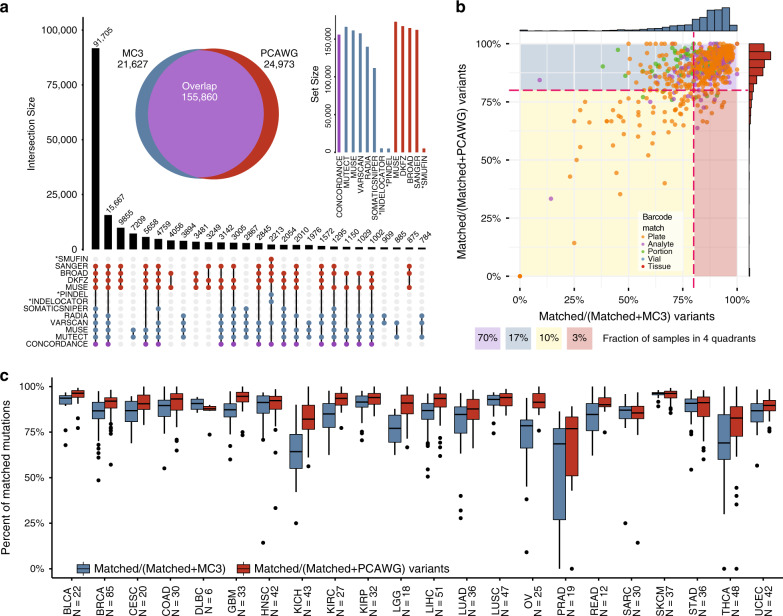
Landscape of mutations overlap by caller, sample and cancer type. **a** UpSetR^41^ plot shows the variant calling set intersection by caller. The y-axis indicates set intersection size and the *x*-axis uses a connected dot plot to indicate which sets are considered. Only the largest 27 intersecting sets are shown. Two insets of the UpSetR plot highlight a classic Euler diagram (left), which indicates the total number of overlapping mutations. A set-size bar chart (right) illustrates the total number of mutations considered from each caller. The concordance set indicates the agreement between WES and WGS. Indel callers are indicated with an asterisk. **b** A scatter plot shows the amount of concordance by sample by calculating the fraction of matched variants divided by the total number of mutations made by MC3 exome sequencing and PCAWG whole genome sequencing (*x* and *y*-axis, respectively) below the total fraction of samples within each quadrant. Each point within the plot is related to tumor portion data collected from the TCGA barcode ID. **c** As shown above, this box plot separates panel b by cancer types (blue considers all MC3 variants, and red boxes indicate all PCAWG variants). Sample sizes are displayed for each cancer; points indicate samples that extend past 1.5 times the interquartile range; and horizontal bars within each box and whisker indicates median matched mutation fraction.

Limiting our analysis to coding regions with sufficient coverage yielded a total of 202,459 variants (155,859 matched, 21,627 unique MC3 variants, and 24,973 unique PCAWG mutations), with 76.7% in concordance between MC3 and PCAWG and 10.7% and 12.3% being unique in MC3 and PCAWG, respectively (Fig. 2a). Concordance can be further separated into SNPs and indels, with 79% and 57% overlapping, respectively (Supplementary Fig. 4). Variant overlap was further investigated to reveal its association with mutation caller, sample, and cancer type (Fig. 2a–c). Consensus variant calling showed 91,705 (45.3%) concordant variants were captured in the intersection of Sanger, MuSE, DKFZ, and Broad callers from PCAWG, as well as Varscan, SomaticSniper, Radia, MuTect, and MuSE callers from MC3. Notably, an additional 7.7% were identified by all SNV mutation detection algorithms, except SomaticSniper. The reduced sensitivity of SomaticSniper is related to its algorithmic consideration of tumor contamination in the matched normal (e.g., skin) for liquid tumors^8^. After optimizing for filtering strategies, we performed a sample level comparison and found that 70% of samples had greater than 80% mutation concordance across the two cohorts. An additional 20% of samples had greater than 80% mutation recoverability in one or the other technique (Fig. 2b). Skin Cutaneous Melanoma performed the best among all cancer types and had the highest variant-matching rates for both MC3 and PCAWG (Fig. 2c). Generally, when considering all MC3 and all PCAWG mutations separately, we observed that PCAWG variant matching rates were generally higher, especially for Kidney Chromophobe (KICH), Brain Lower Grade Glioma (LGG), Ovarian Serous Cystadenocarcinoma (OV), Rectum Adenocarcinoma (READ), and Thyroid Carcinoma (THCA). The differences in OV are likely driven by whole genome amplified library preparation. Generally, the median fractions for matching MC3 variants were lower than those of matching PCAWG variants. This result was unexpected because MC3 provided fewer unique variants overall, suggesting that a large fraction of PCAWG unique variants reside in a few samples. Furthermore, after accounting for hypermutators, we identified a correlation between non-silent mutations per megabase and mean consensus percentages at the cancer level in both PCAWG consensus percentages (Mann–Whitney *p*-value = 1.97 × 10^−3^) and MC3 consensus percentages (Mann–Whitney *p*-value = 6.59 × 10^−4^, see “Methods” section, Supplementary Fig. 1c, d). Despite strong rank statistics, neither set exhibited strong correlation values for MC3 variants or PCAWG variants, *R*^2^ statistics = 0.31 and 0.17 respectively with the majority of cancer types exceeding 80% mean concordance. Thus, one may expect to observe slightly higher variant fidelity in samples with more mutations.

### Variant allele fraction affects call-rates

After achieving a comparable data set and merging MC3 and PCAWG variants, we found that low VAF is the prevailing attribute of unique mutations. VAF is a fundamental factor in somatic variant detection, as well as sub-clonal structure prediction, and is used to predict subclonal tumor growth rates and metastatic potential. To explore the contribution of VAF, we sought to distinguish the contribution of subclonal structure and statistical chance when exploring private mutations in a single call set. We articulate our findings in six broad categories: modeling sequence noise, departure from idealized behavior, sub-clonal modeling, annotation differences, variant-caller effects, and analysis correlations.

### Association of variant allele fraction with recoverability

**Fig. 3:**
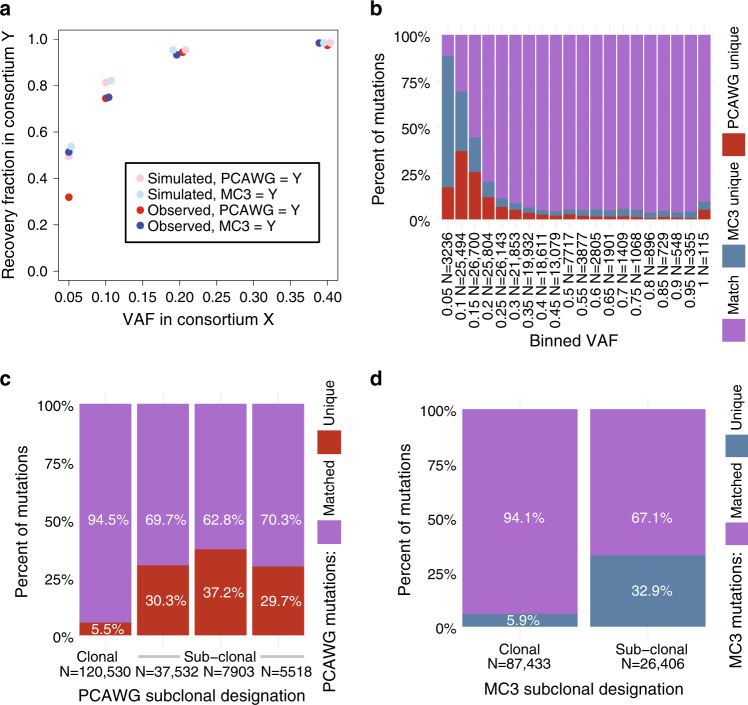
Recoverability simulation and effects of subclones on mutation concordance. **a** Observed recovery rate of PCAWG variants in MC3 (red) and of MC3 variants in PCAWG (blue), alongside sequencing noise simulations calculated from random draws of a binomial model that incorporates the VAF and estimated read depth at each site (light red simulates PCAWG recoverability of MC3 variants, and light blue simulated MC3 recoverability of PCAWG variants). *Y*-axis is described with legend. *X*-axis displays VAF of the comparative data set in regard to *Y*. **b** A stacked bar chart displays the proportion of matched and unique variants (*y*-axis) for different VAF bins (*x*-axis). 180 variants did not provide read count information and were removed from this figure. **c** Stacked proportional histogram shows the fractions of PCAWG matched mutations (purple) and PCAWG-unique mutations (red). Mutations were restricted to SNVs, and subclonality predictions are indicated as either ‘Clonal’ or ‘Sub-clonal’. Columns 2–4 reflect sub-clonal assignment provided by PCAWG (Note: only a few samples reported five predicted subclones and were not included in this analysis). The number of variants represented for each clonal assignment is shown on the *x*-axis. **d** Similar to panel **c**, a stacked proportional histogram illustrates the proportion of matched and unique variants for MC3 which provide estimates of total number of matched or unique variants called by MC3.

We have observed that variants with low VAF are less likely to be reported in both call-sets. This finding relates to the lower sensitivity of somatic variant callers for variants with low VAF. To illustrate this principle, we estimated the expected overlap rate between MC3 and PCAWG at different VAFs. The sensitivity of MuSE across a range of VAFs and read depths in synthetic data was reported in Fan et al., 2016^3^. We used these reported benchmarking characteristics of MuSE to estimate the expected overlap rate between the MuSE call-sets of MC3 and PCAWG across a range of VAFs (see “Methods” section). These expectations, which involve lower overlap rates at lower VAFs, generally tracked observed data but tended to overestimate observed overlap rates, especially for predicting the recovery fraction of MC3 variants in PCAWG. (Fig. 3a) The discrepancies between expectations and observations may relate to simplifying assumptions that made this modeling possible (see “Methods” section).

More generally, we observed that VAF had a greater association with variant recovery rates than predicted by the binomial model (Fig. 3b). A random forest regression model trained on five statistics characteristics of VAF distribution per PCAWG sample and another five for that of the corresponding MC3 call-set predicted the fraction of variants per sample unique to PCAWG with 0.85 (0.86—when restricting to variants called by MuSE) Spearman correlation of test-set observations and a 0.68 (0.78) coefficient of determination (R^2^).

The strong association of VAF with recovery rates by call-set, despite modest explanatory power of the binomial, indicates important departures from idealized behavior. These departures could include explanations such as: PCR amplification violates the assumption of independence of reads, imputed read depths are systematically inflated, or some low-VAF variants represent sequencing artifacts. We conclude that non-ideal effects of VAF predict the majority of sample-level variance in fraction of co-called variants.

### Exploring subclonality

**Fig. 4:**
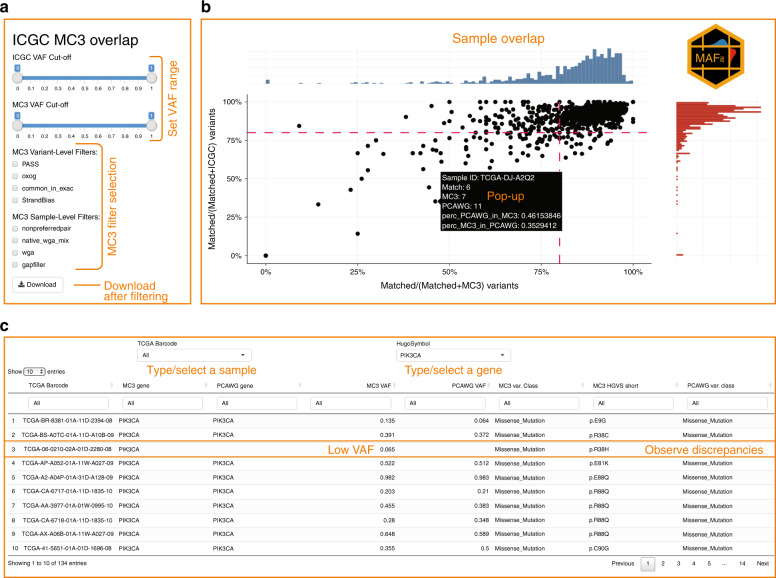
Screenshots of online tool MAFit. Here we display screenshots from the MAFit on-line interface. Currently there are three main components to the interface: **a** A side panel shows sliders and radio buttons to filter data set to remain inclusive. In addition, a download button is available that will download the underlying data table. **b** MAFit rebuilds Fig. 2b in the first tab of the on-line interface. Each alteration to the radio buttons or VAF sliders will result in an updated figure. In addition, if one’s hovers over a point on the scatter plot, a pop-up window will automatically display, providing the user with basic statistics used to calculate that point, i.e., total number of mutations, number of unique and matched mutations. **c** A table is also presented based on the selection criteria in panel **a**.

One possible explanation for some variants being private to one call-set is that the sequencing aliquots for the two sequencing projects came from subclonally-distinct microregions of the same tumor. To investigate this possibility, we tested whether the MC3 and PCAWG call-sets differed from each other systematically at the subclonal level (Fig. 3c, d). We hypothesized that tumors with a more complex subclonal structure (i.e., greater number of subclones) would have larger systematic differences in the VAF of shared variants between the MC3 and PCAWG call-sets. We found a small but highly significant effect: each additional subclone increased the average absolute difference in VAF of the shared variants between MC3 and PCAWG by 0.003, with a *p*-value of 1.3 × 10^−11^ (linear regression); this effect reversed after controlling for tumor purity, indicating that the observed trend does not provide evidence of this interesting concept in re-sequencing (see “Methods” section for details). We do not have evidence that systematic VAF differences between call-sets of the same underlying sample associate with tumor heterogeneity. Real time effects of VAF differences between these two data sets can be observed using the online MAFit tool (Fig. 4).

### Annotation differs by call-set

Genome annotation is critical for biological interpretation and downstream analysis of sequencing data. In order to avoid issues that arise from annotation differences, we only considered genomic locations in our intersection strategy. In doing so, we observed 2153 annotation differences where MC3 and PCAWG had different genes annotated for the same mutation. After restricting the mutation type to missense mutations and indels, 789 annotations differences remained. Most of these had the same mutation types annotated by both call-sets (690 SNPs, 15 insertions, 50 deletions), but some discrepancies remained. Notably, 413 out of 789 mismatch variants are labeled coding in MC3 but non-coding in PCAWG (Supplementary Data 4). We also observed four mutations that were annotated as cancer gene mutations by MC3, but as non-cancer gene mutations by PCAWG, and another four mutations that were annotated as cancer gene mutations by PCAWG, but as non-cancer gene mutations by MC3. One such example subsumed two mutations on chromosomal location 3p21.1 (genomic locations chr3:52442525 and chr3:52442604) that were annotated as missense mutations of *BAP1* by MC3, but as 5’Flank SNPs of *PHF7* by PCAWG. While identical pipelines resolve such differences, we stress the potential for misinterpretations when combining these publicly-available datasets.

### Effects of software

Another important issue we assess is the degree to which differences in bioinformatics pipelines impact concordance. We extracted calls from MuSE and MuTect, both of which were executed on each dataset, and examined 6 subsets of results: MuSE-only-calls and all calls save MuSE-calls (the complement), MuTect-only-calls and their complement, and MuSE + MuTect calls and their complement. MuSE and Mutect each generate around 95% of the total calls, of which each respective subset shows close to 80% concordance between WES and WGS (Supplementary Fig. 5). These call sets themselves overlap almost completely, with their combination (MuSE + MuTect) giving a marginally higher concordance. Conversely, the data-specific caller combinations (referred to above as the complements) each furnish small call sets which vary considerably between WES and WGS (concordance as low as 15%). Because of the vast difference in the sizes of the MuSE/MuTect and the complementary call sets, there is little difference in the original analysis versus analyses restricted to variant callers common to both platforms. Differences in software pipelines do not appear to be significant confounding factors in concordance here.

### Effects on higher-level analysis

We also sought to assess how higher-level analyses might be impacted using mutation signature analysis as a representative. We ran SignatureAnalyzer28 to ascertain signatures between matched WGS and WES samples for each case. A total of 563 of 739 cases (76%) showed the same dominant signature between WES and WGS and the multi-element signature vectors for each case are very highly correlated with one another, the average Pearson coefficient being almost 90%, with a cohort significance of <2 × 10^−6^ (Fisher’s Test, “Methods” section, Supplementary Fig. 6). These observations suggest that signature analysis is relatively insensitive to data type when concordance is high, as it is here.

### Landscape of private WES and WGS mutations

After identifying many possible sources of variation among private variants, we sought to characterize the fraction of variation explained by previously identified factors (Supplementary Fig. 7, see “Methods” section). As displayed, subclonal and low VAF variants make up the largest fractions of explained variants for private MC3 and PCAWG variants. Notably, for private MC3 calls, indels (not called by MuSE or MuTect) are the next highest source of variation explained. GC-content and poor performing cancers such as THCA, KICH, and PRAD make up a smaller portion of the total number of private mutations.

### Variants present in only one public call-set

We sought to classify cancer driver mutations uniquely identified by MC3. After removing two outlier samples having excesses of unique mutations (TCGA-CA-6717-01A-11D-1835-10, TCGA-BR-6452-01A-12D-1800-08), we observed 424 mutations in cancer genes^28^ (median read depth = 97, median alternative allele count = 9) The four most frequently mutated genes were: *KMT2C* (22-mutations), *PIK3CA* (12), *SPTA1* (9), and *NCOR1* (9). Interestingly, the majority of unique *PIK3CA* mutations not identified by PCAWG were at 2 locations: E542K/G (5), and E545K (4). Whether this phenomenon reflects technical bias of WGS or is a product of subclonality warrants further investigation.

The MC3 effort produced two mutation files: one controlled access somatic mutation file that represents nearly all mutations found by all callers, and a second was modified by the scientific community for public use. There are two critical differences in these files involving the reporting of mutations in exonic regions and mutations reported by a single variant caller. Since we limited our analysis strictly to exonic regions, we observed that 92% of the 9138 PCAWG private mutations found in the MC3 controlled access file were only identified by a single variant caller (Supplementary Fig. 8). As expected, the highest unique variant caller overlap was observed in MuTect and MuSE, two tools that were used by both MC3 and PCAWG. This observation accounts for 30% of PCAWG private variants.

We investigated how many variants unique to the MC3 somatic public access call-set could be found in the PCAWG germline call-set for the same patients. We identified a total of six such variants (each in a different sample), five of which were flagged in the MC3 public call-set with one filter or another. Overall, this indicates that variants that have been incorrectly designated as germline or somatic are an extremely uncommon source of variation between the two projects.

### Variants in GC-extreme intervals

**Fig. 5:**
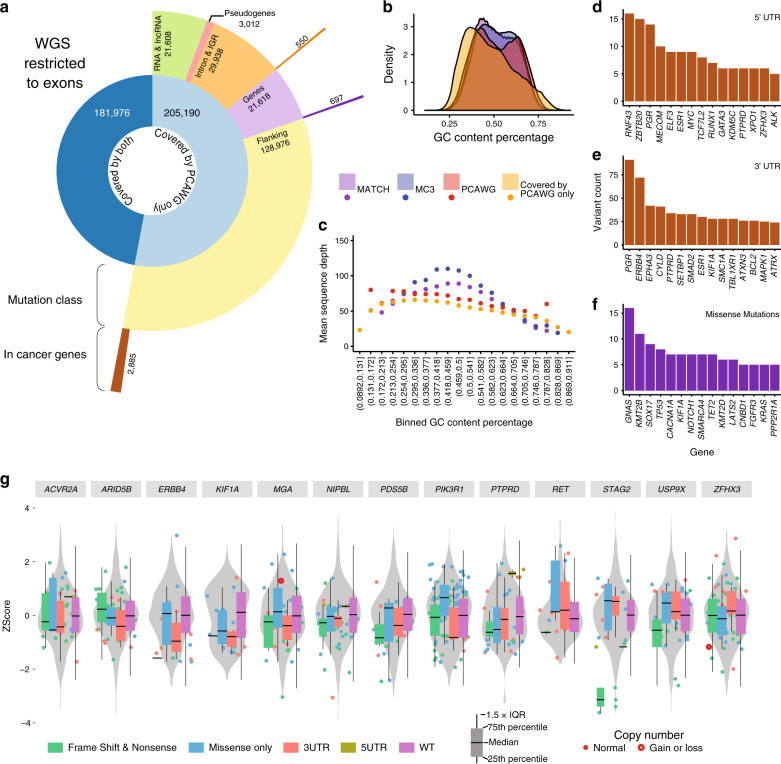
WGS mutations in exonic regions not captured by WES. **a** A sunburst diagram provides a breakdown of variants that are removed during the coverage step of the tool. The innermost circle represents the total number of variants identified upon filtering for exome beds used by MC3. Then, we restrict PCAWG variants to well-covered MC3 regions for each sample. The majority of gencode.v19 annotated and the BROAD target bed file of exonic regions are sufficiently covered by PCAWG in flanking regions: 3’UTRs, 5’UTR, and 5’Flanking. The outermost ring illustrates the number mutations identified by PCAWG that were poorly covered by MC3. **b** A density plot illustrates the density of percent GC-content from a 100 bp window surrounding a variant. Four variant-sets are displayed: matched, private to MC3, private to PCAWG, and we extend our dataset to include exonic variants not covered by WES but sufficiently covered in WGS (Covered by PCAWG only). **c** A scatter plot displays mean sequence depth (*y*-axis) by increasing GC-content bins (*x*-axis). Points are colored according to variant set (same as panel **b**). **d**–**f** Total annotated mutations counts from 3 different annotated regions are shown for 5UTR, 3UTR, and missense mutations, respectively. **g** Expression *Z*−Scores for 3’UTR using all TCGA-UCEC samples. *Cis*-RNAseq expression violin plots are displayed for 13 genes. On top of the gene-level distribution violin plot, box and whisker plots display sample expression based on mutation classification (box include 25th quantile to 75th quantiles, and whiskers extend to 1.5 times the interquartile range).

Since it is well-known that the efficiency of exome capture is adversely affected by very high or very low GC-content^29,30^, we sought to test whether GC-content was associated with call rates in MC3 and PCAWG. We used a plug-in for VEP^31^ to annotate all matched and private SNVs with CADD^32^ in order to annotate each variant with the percentage of the neighboring 100 bases that are a G or C. First, we assessed how the distribution of read depth across GC-content changes between MC3 and PCAWG (Fig. 5b). PCAWG was found to have a fairly uniform read depth across GC-content bins, while MC3 read depth was concentrated in regions of moderate GC-content (Fig. 5c). The low read depth in MC3 at regions of extreme GC-content was in turn associated with lower variant recovery rates in these regions but did not grossly affect the number of variants recovered by MC3 because regions of extreme GC-content are relatively rare in the genome overall and in exome-capture regions in particular.

An in-depth analysis of these regions revealed that 76 mutations in known driver genes, identified in the combined TCGA data by Bailey et al. 2018, were missed in GC poor (GC fraction < 0.3) or GC rich (GC > 0.7) regions^28^. Three such instances revealed *VHL* mutations in KIRC that were overlooked in GC rich regions of this gene (two of these three recur). In addition, these 3 samples are not reported to carry a *VHL* mutation in the MC3 public data set. Other such instances include 7 *SOX17* mutations, *LATS2* (6), and *CACNA1A* (6). These findings emphasize the advantages of uniform coverage using WGS.

The bases flanking a mutation (tri-nucleotide context) affect mutation rate, which should be approximately equal between MC3 and PCAWG, and also the rate of introduction of sequencing artifacts. Large differences in the call-rates of MC3 and PCAWG and particular nucleotide sequences could indicate a sequencing artifact unique to one or the other call-set, which might arise from different procedures for computationally filtering or biochemically preventing sequencing oxidation products. Therefore, we sought to test whether the trinucleotide context of variants correlated with relative call-rates in MC3 and PCAWG. Before applying the MC3 OxoG filters, we found a huge predominance of CA variants unique to MC3, with the trinucleotide contexts most specific to one database or another being 7-9 times more specific than the least specific trinucleotide contexts. After applying the MC3 OxoG filters, nucleotide contexts differed by less than four-fold in their specificities. The residual differential specificity by trinucleotide context after filtering can either indicate differences in sequencing artifact abundance and filtration by project, or could merely be a consequence of the fact that nucleotide context is also correlated with VAF and the distance from transcription start sites, which may independently affect MC3 and PCAWG relative call-rates.

We extended the nucleotide context and performed mutation spectrum analysis, comparing all MC3 and all PCAWG mutations found after restricting the two data sets to exonic regions as described above (Step 3 of Fig. 1a). We then calculated transition and transversion frequencies in each cancer type. After removing hypermutated samples and OxoG artifacts, we used a chi-squared test to determine the similarities and differences between cancer types in the full exome space compared versus the captured exome space. Strikingly, we did not identify significant differences in mutation spectrum in the majority of cancers. We did observe significant differences (FDR < 0.05) in the mutation spectrum for COAD, KICH, LUAD, and OV (Supplementary Data 5). These observations included strong discrepancies for AG and CG transition differences in KICH and OV, respectively. AT and CA transversions contributed mostly to COAD and LUAD differences (Supplementary Fig. 9). While these differences may reflect sequencing artifacts, such as whole genome amplified DNA in OV or low sample size, we believe the data can still provide more information pertaining to additional cancer genes and oncogenic mechanisms.

### Non-Coding/Flanking intersections with low coverage

With the growing use of WGS in many labs, we sought to identify which mutations are gained by extending to this form of data. One major observation from our pipeline highlighted that some variants in exome regions were not well covered by WES (Fig. 1a Step 3). Using this mutation set we investigated the most recurrent members as derived by WGS but not by MC3 in exonic regions as defined by gencode.v19 (Fig. 5a). We observed 697 mutations in cancer genes^28^ uniquely called by WGS (Supplementary Data 6). We defined flanking mutations as all non-translated mutations near exons, i.e., 5’UTR, 3’UTR, 5’Flanking, and 3’Flanking regions, as they make up the majority of mutations not present in the MC3 public MAF. Recurrent mutation analysis identified the most frequently mutated genes in 5’UTR (Fig. 5d), 3’UTR (Fig. 5e), and missense mutations (Fig. 5f). We found the most frequently mutated 3’UTR in cancer genes was *PGR* (91 mutations allowing for multiple mutations per sample), followed by *ERBB4* (72), *EPHA3* (42), *CYLD* (41), and *PTPRD* (34). To extend this analysis, we used RNAseq data collected by TCGA to determine mutation type specific *cis-*expression patterns, which clearly shows correlation of UTR mutations on RNA abundance (Fig. 5g).

**Fig. 6:**
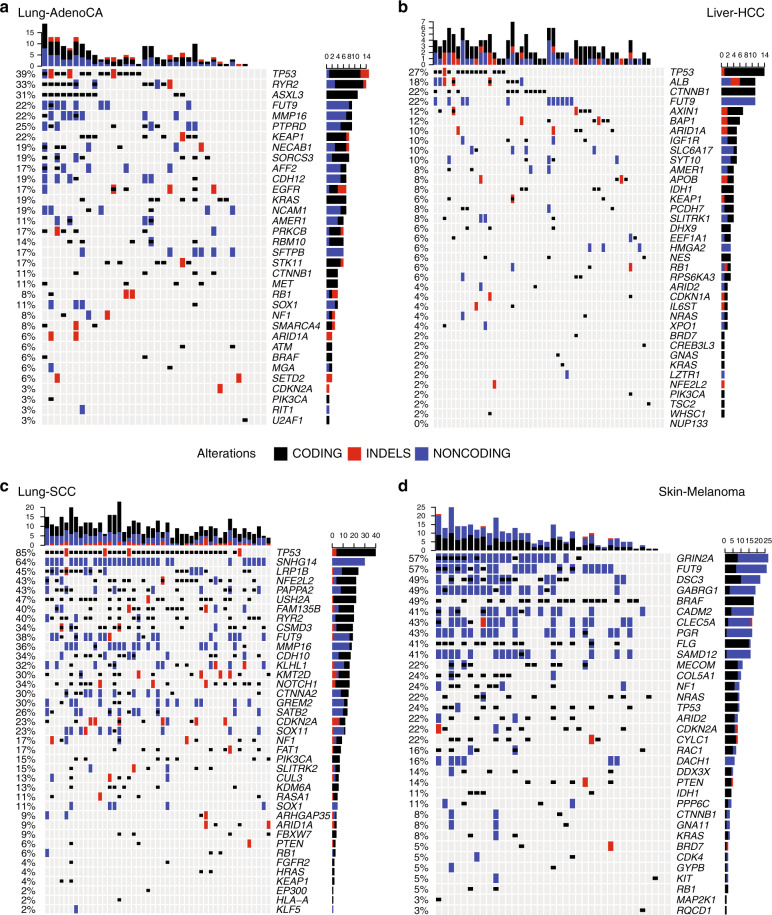
Significantly mutated gene analysis with the inclusion of UTR mutations. OncoPrint plots were generated using the R package ComplexHeatmap^42^ for four cancer types: LUAD (**a**), LIHC (**b**), LUSC (**c**), and SKCM (**d**). We report all SMGs identified by Bailey et al. 2018^28^, as well as top significantly mutated gene hits from MuSiC that include non-coding mutations. While many non-coding mutations look promising, further investigation yielded little support for driver identification status.

Finally, similar to previous studies^33,34^, we investigated the potential effect of non-coding mutations when determining significantly mutated genes (SMG). Using MuSiC^35^ with the no-skip-non-coding option, we rescued non-coding mutations annotated by PCAWG and included them in the significantly mutated gene (SMG) analysis. We only performed SMG analysis on cancer types having greater than 35 samples (BRCA-Breast-AdenoCa, HNSC-Head-SCC, KICH-Kidney-ChRCC, LIHC-Liver-HCC, LUAD-Lung-AdenoCA, LUSC-Lung-SCC, SKCM-Skin-Melanoma, STAD-Stomach-AdenoCA, THCA-Thy-AdenoCA, and UCEC-Uterus-AdenoCA). We initially identified potential driver-gene candidates (*FUT9*, *MMP16*, *SNHG14*, and *SFTPB*, Fig. 6) not previously reported in Pan-Cancer whole genome analysis, but further investigation did not support these candidates with the exception of *SFTPB*.

*SFTPB* (FDR 1.56e−07) in LUAD was recently reported to be significantly mutated using a larger set of these same data^34^. As reported, this gene is involved in a lineage-defining surfactant protein. While six mutations contributed to its SMG status, only 1 3’UTR mutation was reported for LUAD in the MC3 controlled data set. Furthermore, this single indel was only found by one variant caller (Varscan). We confirmed the impact of *SFTPB* UTR mutations by performing a genome-wide association analysis of expression differences and found that samples with *SFTPB* mutations showed lower RNA abundance in *PCDHA7*, a gene known to be involved in cells’ self-recognition and non-self-discrimination (chi-squared *p*-value 3.6 × 10^-8^). While other promising candidates exist, such as *FUT9*, a fucosyltransferase involved in organ bud progression during embryogenesis and has been implicated in cancer initiation^36^, we found no additional evidence for supporting its driver status.

## Discussion

The research community is increasingly leveraging technology advances to integrate data at larger scales. We performed a comparative evaluation of ~750 samples with joint exome and whole genome sequencing mutation calls provided by two consensus mutation calling efforts, MC3 and PCAWG. This joint data set is encouraging, suggesting that ~80% of the predicted somatic mutations were captured by both efforts. Furthermore, a combined 90% of samples have greater than 80% variant concordance when considering covered exonic mutations from individual cohorts separately. Analysis of this data set also revealed three major contributors to variant discrepancies: (1) low variant allele fraction, (2) variant filtering decisions, and (3) technological limitations. Software differences were not an appreciable confounder.

Distinct advantages and disadvantages accompany somatic mutation calling when utilizing captured WES or WGS. We found that ~70% of the discrepancies between whole genome and whole exome sequencing are influenced by low variant allele fraction. This information holds many implications in identifying subclonal heterogeneity in the tumor of interest. Other discrepant calls originate from the decisions made on how to filter and distribute publicly available mutation calls. Higher-order mutation signature analysis does not appear to be inordinately affected by these differences. We show that reported germline variants were negligible, but nearly 30% of the private PCAWG mutations were not reported by MC3 because only a single variant detection algorithm identified them. We want to emphasize that, while somatic variant detection in cancer is commonplace, there are still many issues to reconcile.

Finally, we found additional mutations only observable in exonic regions using either WES or WGS. For example, WES uniquely identified 424 mutations in cancer genes with median VAF of ~10%. We also highlight ~700 WGS mutations from cancer genes, of which ~10% are attributable to regions of high and low CG-content; thus, highlighting the advantages of more uniform coverage from WGS.

Only about 2% of the genome is protein coding. For the last dozen years, cancer genomics has provided a comprehensive molecular characterization of many different tumor types, thanks in large part to The Cancer Genome Atlas and other publicly funded efforts. The community is just starting to explore how exomics, transcriptomics, proteomics, and methylomics can be woven together across this 2% of the genome. We anticipate a general transition from WES to WGS, but this analysis is meanwhile reassuring that few clerical mutations were overlooked in WES and that WGS is capable of recapitulating previous genomic findings.

## Methods

### Human research participants

The Cancer Genome Atlas (TCGA) collected both tumor and non-tumor biospecimens from human samples with informed consent under authorization of local institutional review boards (https://cancergenome.nih.gov/abouttcga/policies/informedconsent).

### Sample overlap

TCGA barcodes carry metadata that reflect tumor portions and different aliquots. As noted in Fig. 1b, TCGA barcode differ slightly in the comparison between MC3 and WGS aliquots. A brief description of the breakdown of the TCGA barcode is outlined below.

Example: TCGA-DD-0001-01B-01D-A152

- TCGA-Project
- DD-Tissue source site: the tissue location of tumor that matches clinical metadata.
- 0001-Participant code
- 01-Sample type: i.e., solid tumor (01), primary blood derived tumor (03), solid tissue normal (11), blood derived normal (10)
- B-Vial: the order in a sequence of samples, i.e., A = first in sequence, B = second in sequence
- 01-Portion: sequential order of the 100–120 mg of samples
- D-Analyte: molecular analyte type for analysis, i.e., D for DNA and W for WGA.
- A152-Plate: sequential location of a 96-well plate

A lookup table outlining these fields is located at the GDC: https://gdc.cancer.gov/resources-tcga-users/tcga-code-tables. In order to determine the role of aliquot differences in assessing mutation concordance, we re-analyzed the clonality and overall mutation overlap after stratifying for exact barcode differences. We observed that the effect of matching barcodes on match variant frequency has little effect.

### Assessing cancer subtype selection preference

Analysis working groups for TCGA projects were primarily subdivided according to cancer types. Scientific experts gathered in consortia from around the country to participate in characterizing many tumors using high throughput data generated on many substrates, e.g., WES, RNAseq, etc. At the conclusion of these projects, groups were asked to hand-select a subset of samples to perform validation sequencing (WGS, the samples used in this analysis). The selection criteria differed from group-to-group and sometimes resulted in an overabundance of one subtype over another. To determine cancer subtype selection bias, we performed an enrichment analysis. Using clinical data we calculated (for each cancer type) the subtype fraction in the WES cancer cohort and measured whether the fraction was similar to WGS set of samples using a Fisher’s exact test.

### Defining exonic regions

We used the same definition as Ellrott et al. to reduce whole genome and exome calls to define genomic coordinates^27^.

### Coverage calculations

Fixed-step (step = 1) Wiggle coverage files (.wig) for both MC3 and PCAWG were provided by the Broad Institute. The wig format is a binary readout of sufficient sequencing coverage for genomic data. Here, sufficient coverage is defined as bases with 8 or more reads at a given location. These wig files were processed and reduced to exonic regions using the wig2bed function from BEDOPTS^37^.

After the preliminary screen of coverage-reduced MAF files, we observed that matching mutations (identified by PCAWG and MC3) were removed from one technology and not the other after the coverage reduction step. To account for this issue, we performed a self-coverage reduction step to that identified 6460 mutations. We describe some properties of those mutations here. The median tumor depth reported by MC3 from these variants is 12 reads (+/− 3 median absolute difference). The median tumor depth reported by PCAWG in this region is 39 reads (+/− 35.6 median absolute difference), suggesting wide variance of tumor read depths that were removed. However, the mode tumor depth of the PCAWG variants was 13, justifying this removal of variants with low read support. Finally, we determined how many of these poorly-covered variants originated from cancer driver genes. We observed 126 mutations from the MC3 file, and 156 cancer mutations were eliminated at this stage in the comparison.

### Overlapping mutations

After reducing the variants to be within exome sequencing target region, within same exon definitions, and having enough sequencing depth, the remaining variants from ICGC and PCAWG were stored in a SQLite database to enable fast lookup. We then executed a full join between the two sources of variants by matching the donor ID, sample ID, and the genomic range of each variant. The full join output was further cleaned up to remove duplicated filters due to naming variations and duplicated variants due to DNPs.

- Matching IDs
- Matching chromosomes
- End position greater than or equal to start position
- Start position is less than or equal to end position.

### Deduplication of variants

After merging the PCAWG and MC3 data, we observed different strategies were taken by MC3 and PCAWG to capture neighboring variants, i.e., complex indels, di-nucleotide (DNP) and tri-nucleotide (TNP) polymorphisms. To address complex indel events (SNVs in indel regions), the MC3 working group absorbed the variants made by SNV callers into the assignment made by Pindel. Conversely, PCAWG merged DNP and TNP events into a single event. These strategies resulted in many duplication events from MC3 and PCAWG: 1731 and 62, respectively. These events encompassed 3457 and 119 events, respectively. To address these differences, we merged PCAWG variants into MC3s complex indel events, and MC3 variants into single DNP or TNP events.

### Filtering optimization

After reducing the starting pool of possible mutations from 746 samples to covered exons, we sought to identify the optimal set of MC3 filters that provide the largest number of samples with greater than 80% concordance from the two technologies with the simplest schema. This was performed comprehensively using all possible combinations of filters, often with more than one filter per variant, with the MC3 cohort (131,071 filter combinations). Filter flags include: “common_in_exac”, “gapfiller”, “native_wga_mix”, “nonpreferredpair”, “oxog”, “StrandBias”, and “wga”. We pre-defined the exclusion of variants in MC3 flagged as OxoG along with the inclusion of all PASS variants. The comprehensive filter analysis resulted in two major clusters of variant recoverability (Supplementary Fig. 3). Here, we observed the computational trade-off of identifying more matched variants at the cost of more unique MC3 calls. Below, we highlight five strategies considered for analysis (Supplementary Data 2).

1. Only consider variants labeled PASS by the MC3 filter column.
2. Only remove variants labeled OxoG by MC3.
3. Prioritize G1 (samples in the most recoverable quadrant, MC3 and PCAWG samples with greater than or equal to 80% from both efforts.)
4. Prioritize total number of matched variants.
5. Maximize total number of samples in the most recoverable quadrant (Fig. 2b) while maximizing the difference between unique MC3 variants and matched variants thus generating fewer unique calls.

After considering complexity, we chose to move forward with strategy 2 for the entirety of this study due to its simplicity and relative similarity to other filtering schemes. We recognize that by selecting a single filtering strategy, we are limiting the data slightly and likely introducing some false positive variant calls. However, this strategy allowed us to maintain larger sample sizes and to capture ~15,000 more matched variants than the PASS only strategy at the cost of ~3500 unique mutations calls for MC3.

### Assessing mutations per megabase and cancer type concordance

Mutations per megabase data were collected from the broader TCGA dataset and reduced following the same methods outlined previously^28^. Briefly, this systematically removed hypermutators from the dataset. This resulted in a set of 625 samples from the MC3/PCAWG dataset studied here and 8852 TCGA samples. Both Pearson and Mann-Whitney correlations statistics were performed to assess the association of non-silent mutations per megabase and concordance statistics.

### Simulation of sequencing noise and recoverability

Fan et al. benchmarked the sensitivity of MuSE at recovering somatic variants across 24 combinations of VAF and read depth^3^. When simulating the recovery of PCAWG variants in MC3 we assumed that the VAF observed in PCAWG was the true VAF. We matched the observed VAF of each variant to the closest VAF reported in Fan et al.

For our analysis, the best value to use as the read depth when predicting the MC3 recovery rate of PCAWG variants would be the MC3 read depth at the same site and sample as the PCAWG variant. However, it was not practical to obtain MC3 read depths at sites without MC3 variants, so instead we simulated an MC3 read depth for each PCAWG variant by randomly sampling from the read depths of observed MC3 variants from the same sample as the PCAWG sample. We then matched these simulated read depths for each variant to the closest read depths reported in Fan et al.

For the binned VAFs and read depths for each PCAWG variant obtained as above, we pulled the corresponding sensitivities of MuSE from the Fan et al. paper and simulated MC3 variants with probability equal to these sensitivities.

### Integrating clonality

Both consortia considered clonality in their comprehensive characterization of the somatic mutations. Locations of these files are provided in the data availability section. Here, we provide a brief summary of the strategies used to compile these resources. First, the PanCancer Atlas working-groups used MC3 mutations to predict subclonal structures using ABSOLUTE^38^. This tool uses copy number, recurrent karyotype, and mutation data to calculate copy number purity and cluster identification. Furthermore, the PanCancer Atlas working group only made the distinction of clonal and subclonal mutations and did not attempt to further assign sub-clonal mutations to other likely heterogeneous clusters. PCAWG, on the other hand, used a consensus calling approach incorporating 11 different clustering tools. Here, we evaluated cluster-ID which represents those mutations that are clonal (ID = 1), with other clusters representing mutations that are subclonal (ID = 2 through 4). For this analysis, we restricted our data to SNVs to be consistent with calls made by the PanCancer Atlas calls of MC3 mutations.

### Fraction of private variation explained

In Supplementary Fig. 7 we provide a breakdown of different sources of variant described in our analysis using publicly available data. For MC3 all private variants were classified as into 3 variant types (Indel, MissensePlus, and Other). Specifically, indels are comprised of: “Frame_Shift_Del”, “In_Frame_Ins”, “Frame_Shift_Ins”, and “In_Frame_Del”. MissensePlus variants are comprised of: “Missense_Mutation”, “Nonsense_Mutation”, “Nonstop_Mutation”, “Splice_Site”. And Other variants are comprised of: “RNA”, “3’UTR”, “5’UTR”, “5’Flank”, “Silent”, “3’Flank”, “Intron”, “Translation_Start_Site”.

On the other hand, PCAWG variants were also categorized into Indels, MissensePlus, and Other. Specifically, indels are comprised of: “Frame_Shift_Del”, “Frame_Shift_Ins”, “De_novo_Start_InFrame”, “Start_Codon_Ins”, “Stop_Codon_Ins”, “In_Frame_Del”, “In_Frame_Ins”, “Stop_Codon_Del”, and “Start_Codon_Del”. MissensePlus variants are comprised of: “Missense_Mutation”, “Nonsense_Mutation”, “Nonstop_Mutation”, “Splice_Site”. And other variants are comprised of: “5’UTR”, “RNA”, “5’Flank”, “Silent”, “3’UTR”, “Intron”, “IGR”, “lincRNA”, “De_novo_Start_OutOfFrame”, and “Start_Codon_SNP”.

In addition to the three variant type categories, six additional sources of variation were added to private variants: Subclonal, VAF5, VAF10, MMcomplement, THCA KICH or PRAD, and GCcontents. As mentioned, subclonal variants are tagged if labeled as identified by the TCGA or ICGC consortia. VAF5 tags all variants with less that 5% VAF. VAF10 tags all variants with VAF greater than or equal to 5% and less that 10%. MMcompelement tags all variants not detected by MuSE or MuTect. And finally, GCcontent was calculated as the fraction of G or C nucleotides in a 100 bp window surround a variant. This was calculated using a CADD plug-in to VEP. The GCcontent flag was assigned to a variant if GC fraction was less and 0.3 or greater than 0.7.

### MAFit: online comparison and visualization tool

MAFit (maf Interaction Tool) is a shinyapp^39^ tool to visualize and extract mutations from the intersection of PCAWG and MC3 call sets. It is an interactive and graphical web-based interface built using R Shiny. The interface consists of three panels: a main scatter plot display, a side box of control widgets, and a mutation table in the bottom pane. Any alteration of a control widget will update the scatter plot and the mutation table, enabling very rapid browsing. There is also a button to download the current data set displayed in the scatter plot.

The main panel displays a scatter plot with marginal histograms of mutations grouped by sample. The axes are percentage of matched mutations versus matched plus call-set-unique mutations. Mouse-hovering on a data point initiates a pop-up window showing specific information for this sample, such as TCGA barcode, number of matched mutations, and numbers of mutations unique to each call-set.

The side panel contains two sets of control widgets which can be used to select data based on different criteria. The top control set consists of two sliders to set the VAF cut-offs for each call-set. Both ends of the slider can be adjusted so that users can obtain a desired interval of the VAF. The bottom control set consists of check-boxes of both variant-level and sample-level MC3 filters. If only variant-level filters are checked, all PCAWG-only mutations will be retained; if at least one sample-level filter is checked, mutations from samples that do not have any checked filters flagged (variant-level or sample-level) will be filtered out. Both variant-level and sample-level filters result in the union of mutations with any checked filter will be shown.

The bottom panel displays a table of mutations based on the selection criteria from the side panel. Columns include information on each mutation, such as TCGA barcode, gene name, VAF, variant class, Human Genome Variant Society (HGVS) nomenclature, etc. Users can sort the table by each column. There are two drop-down selection boxes where users can view mutations from a specific TCGA barcode or Hugo symbol. There is also a search bar, which results in mutations that contain the input in any columns.

### Controlled access files

Having worked with both the TCGA and ICGC consortia, we were privy to the controlled access data (all MC3 somatic variant calls and PCAWG germline calls). These data sets allowed for the further interrogation of unique variants called by both groups.

We performed a mutation variant intersection of MC3 controlled access mutations with unique PCAWG variants in the captured exonic regions. These data can be downloaded using necessary credentials from https://gdc.cancer.gov/about-data/publications/mc3-2017.

We intersected the MC3 public MAF with the PCAWG germline call-set, donor-by-donor. Six MC3 somatic variants were identified as germline variants in PCAWG for the same donor. Of these, five were flagged in MC3 as OxoG or non-preferred-pair artifacts, and only one was marked PASS in MC3. This PASS variant had a depth in the matched MC3 normal of well over 100 with no alternate reads. The minimal intersection between the MC3 somatic call-set and the donor-matched PCAWG germline call-set is evidence that germline contamination in MC3 calls is low.

### Assessment of impact on mutation signature analysis

We ran SignatureAnalyzer^40^ on the corpus of WES and WGS samples from our TCGA cases. This tool reports a vector of *J* = 7 normalized weights corresponding to mutational signatures. Once weights were computed, we used COSMIC signatures as a reference in order to synchronize labels of the fractional weights between WES and WGS data for each case to enable proper comparison. We excluded 5 cases in which signatures were not computed for WGS data. Using the synchronized results, we then assessed both the number of cases for which the tool identified the same dominant signature between WES and WGS data and also evaluated the correlation between WES and WGS vectors for each case using least-squares regression. Statistical significance of each correlation was calculated using a 2-tailed *t*-test. Statistical power of each correlation was limited by the paucity of signature weights because the underlying t-statistic is proportional to the square root of *J* – 2. However, because these cases, and therefore their hypothesis tests, are independent, the cohort constitutes multiple tests of the same hypothesis regarding signatures derived from WES and WGS data. Therefore, we combined individual P-values into an overall cohort *P*-value using Fisher’s log-transform. Namely, the transform of negative 2 times the natural log of the product of the *K* = 739 individual *P*-values is, itself, chi-square distributed with 2 *K* degrees of freedom. Using this transform, we found an overall cohort *P*-value of <2 × 10^−6^.

### Reporting summary

Further information on research design is available in the Nature Research Reporting Summary linked to this article.

## Supplementary information

Supplementary Information

Description of Additional Supplementary Files

Supplementary Data 1

Supplementary Data 2

Supplementary Data 3

Supplementary Data 4

Supplementary Data 5

Supplementary Data 6

Reporting Summary

## Data Availability

Somatic and germline variant calls, mutational signatures, subclonal reconstructions, transcript abundance, splice calls and other core data generated by the ICGC/TCGA Pan-cancer Analysis of Whole Genomes Consortium are described in another publication^10^ and available for download at https://dcc.icgc.org/releases/PCAWG. Additional information on accessing the data, including raw read files, can be found at https://docs.icgc.org/pcawg/data/. In accordance with the data access policies of the ICGC and TCGA projects, most molecular, clinical and specimen data are in an open tier which does not require access approval. To access potentially identification information, such as germline alleles and underlying sequencing data, researchers will need to apply to the TCGA Data Access Committee (DAC) via dbGaP (https://dbgap.ncbi.nlm.nih.gov/aa/wga.cgi?page=login) for access to the TCGA portion of the dataset, and to the ICGC Data Access Compliance Office (DACO; http://icgc.org/daco) for the ICGC portion. In addition, to access somatic single nucleotide variants derived from TCGA donors, researchers will also need to obtain dbGaP authorization. Additional links to data resources used for this project can be found using the following urls: MC3 public MAF file (https://api.gdc.cancer.gov/data/1c8cfe5f-e52d-41ba-94da-f15ea1337efc), PCAWG Public MAF file (https://www.synapse.org/#!Synapse:syn7364923), bed files used for exome restrictions (MC3) (https://api.gdc.cancer.gov/data/7f0d3ab9-8bef-4e3b-928a-6090caae885b), bed files used for exome restrictions (THE BROAD) (https://api.gdc.cancer.gov/data/b1e303a5-a542-4389-8ddb-1d151218be75), wiggle files MC3 (https://www.synapse.org/#!Synapse:syn21785741), wiggle files PCAWG (https://www.synapse.org/#!Synapse:syn8492850), clonality files MC3 (https://www.synapse.org/#!Synapse:syn7870168), clonality files PCAWG (https://www.synapse.org/#!Synapse:syn8532460), cancer subtypes and histological data (https://www.synapse.org/#!Synapse:syn4983466), MAFit online comparison tool (https://mbailey.shinyapps.io/MAFit/), GitHub Repo (https://github.com/ding-lab/mc3_icgc_variant_pipeline), side-by-side MC3-PCAWG comparison (MAF-like) (https://www.synapse.org/#!Synapse:syn21041380). The remaining data is available within the Article, Supplementary Information or available from the authors upon reasonable request.

## Code availability

A public GitHub repository (under an MIT opensource license) at https://github.com/ding-lab/mc3_icgc_variant_pipeline furnishes work-flows, scripts, figures, and computational tools used to assess mutation concordance between maf files. For this project we refrained from accessing alignment files, i.e., BAM files or fastq files. Furthermore, due to the memory footprint of mutation and coverage files we have not included them in the repository. Thus, by removing core data files from the repository the software provided supplies the user with processes and decisions, not a fully automated tool for deployment on additional datasets. In addition to our analysis, the core computational pipelines used by the PCAWG Consortium for alignment, quality control and variant calling are available to the public at https://dockstore.org/search?search=pcawg under the GNU General Public License v3.0, which allows for reuse and distribution.
