# Supplementary material for: Retrospective evaluation of whole exome and genome mutation calls in 746 cancer samples
Source: Nat Commun. 2020 Sep 21;11:4748. doi: 10.1038/s41467-020-18151-y (PMC7505971; doi:10.1038/s41467-020-18151-y)
Supplement: Supplementary file 1 — Supplementary Information [file 41467_2020_18151_MOESM1_ESM.pdf]

Supplementary Information

Retrospective evaluation of whole exome and genome mutation calls in 746 cancer samples

Bailey et al.

Supplementary Figure 1

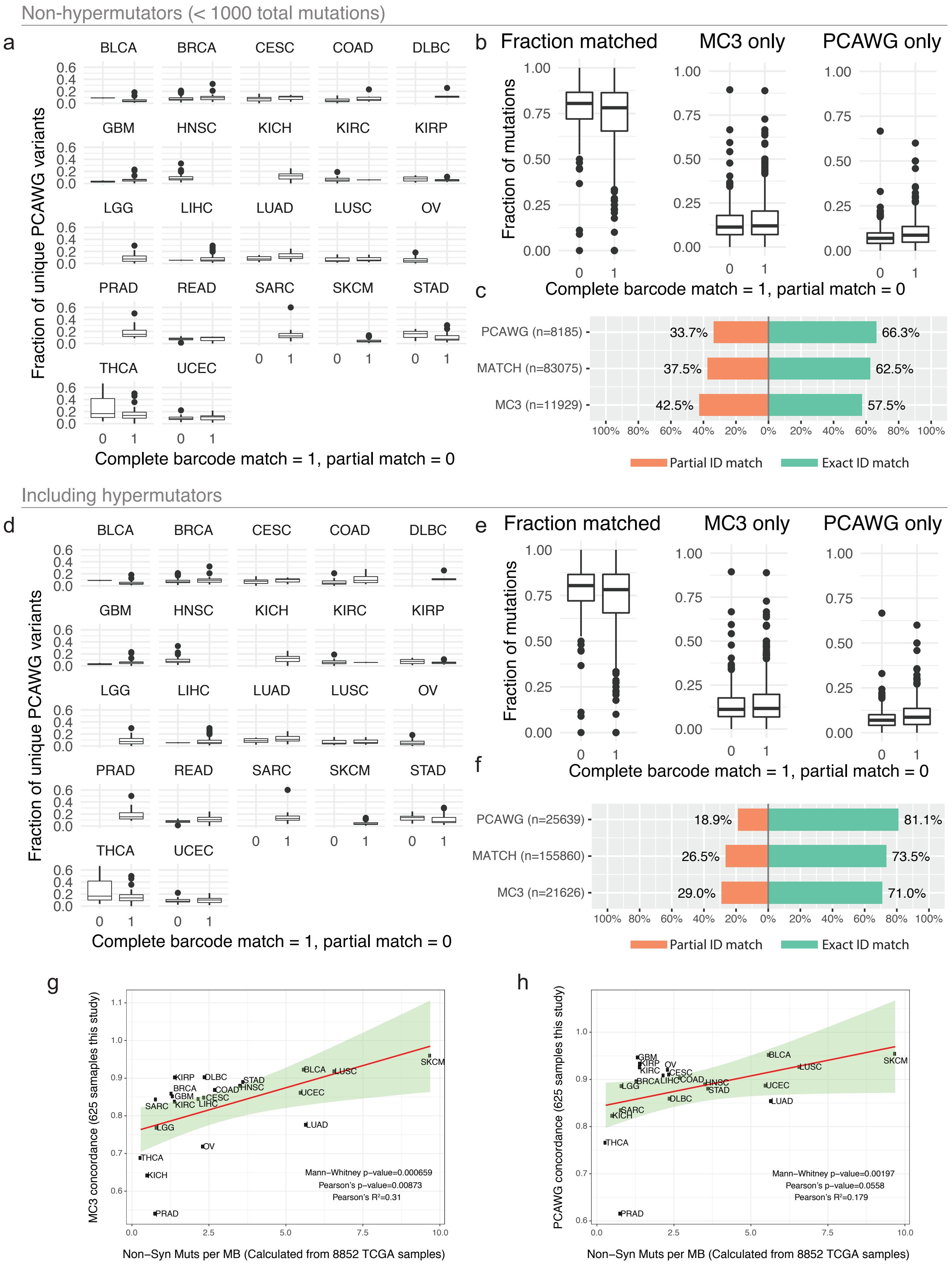

Supplementary Figure 1

Supplementary Figure 1: Effects of exact barcode matching on mutations concordance. a) Boxplots are calculated for fraction of mutations that are unique to the PCAWG data set. The fraction represents the number of mutations per sample, including MATCHED and MC3 unique variant calls. Here, we compare fractions of mutations by cancer types. b) Pan-Cancer fractions for MATCHED, MC3 unique and PCAWG unique variants are shown. Fractions are calculated as above. c) A Likert plot is used to assess the fraction of variants from the same TCGA barcode (same plate location, green color) to other vials, portions, or analytes of the same tumor (orange color). The full bar represents 100% of the fraction and is divided at the midline. The number of variants for each category is shown on the y-axis. Here, only non-hypermutated samples are shown to avoid confounding interpretation. Hypermutators are defined as samples with a total mutation count > 1000; this result shows n=720. d-f) Same as above, but with hypermutated samples (n=744). Points indicated on panels a, b, d, e indicate samples that extend past 1.5 times the interquartile range. g) Correlation plot of MC3 concordance statistics and non-silent mutations per megabase. h) Like the previous panel, this panel illustrates the correlation between PCAWG concordance percentages and non-silent mutations per megabase of the larger TCGA cohort. Green shading indicates 95% CI (panels h and g).

Supplementary Figure 2

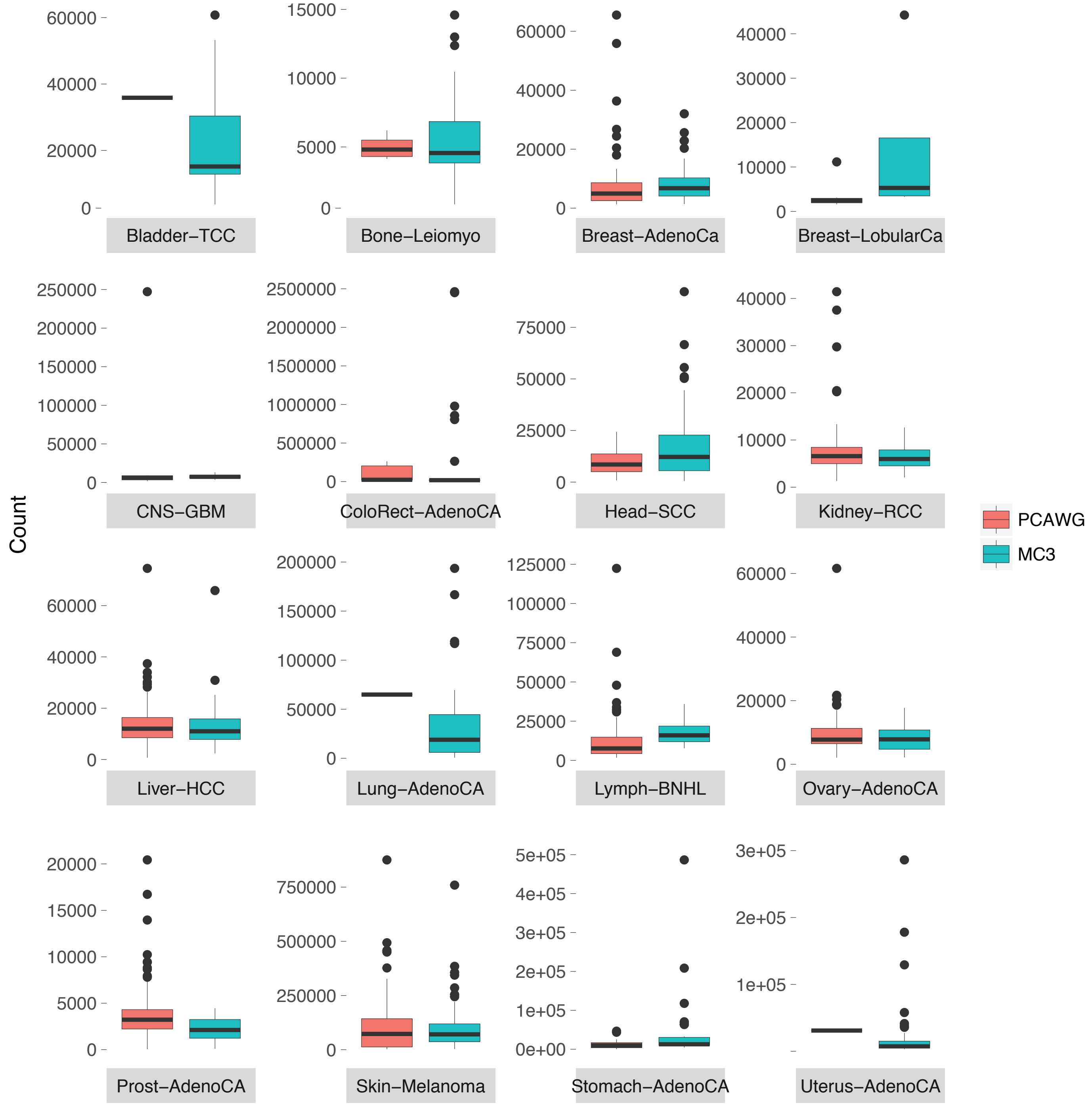

Supplementary Figure 2: Effects of supplementing PCAWG samples with TCGA samples by cancer type. Mutations counts of cancers types split by TCGA and the rest of PCAWG samples. Box-plots show the number of mutations for all PCAWG samples and split by the portion that are also TCGA. Points indicate samples that extend past 1.5 time the interquartile range. Horizontal bars shows median mutation counts. Here we show that mutations counts do not differ greatly between cohorts and that mainly the excess of mutations after sample selection is due to a few outliers and not cancer types selection bias.

Supplementary Figure 3

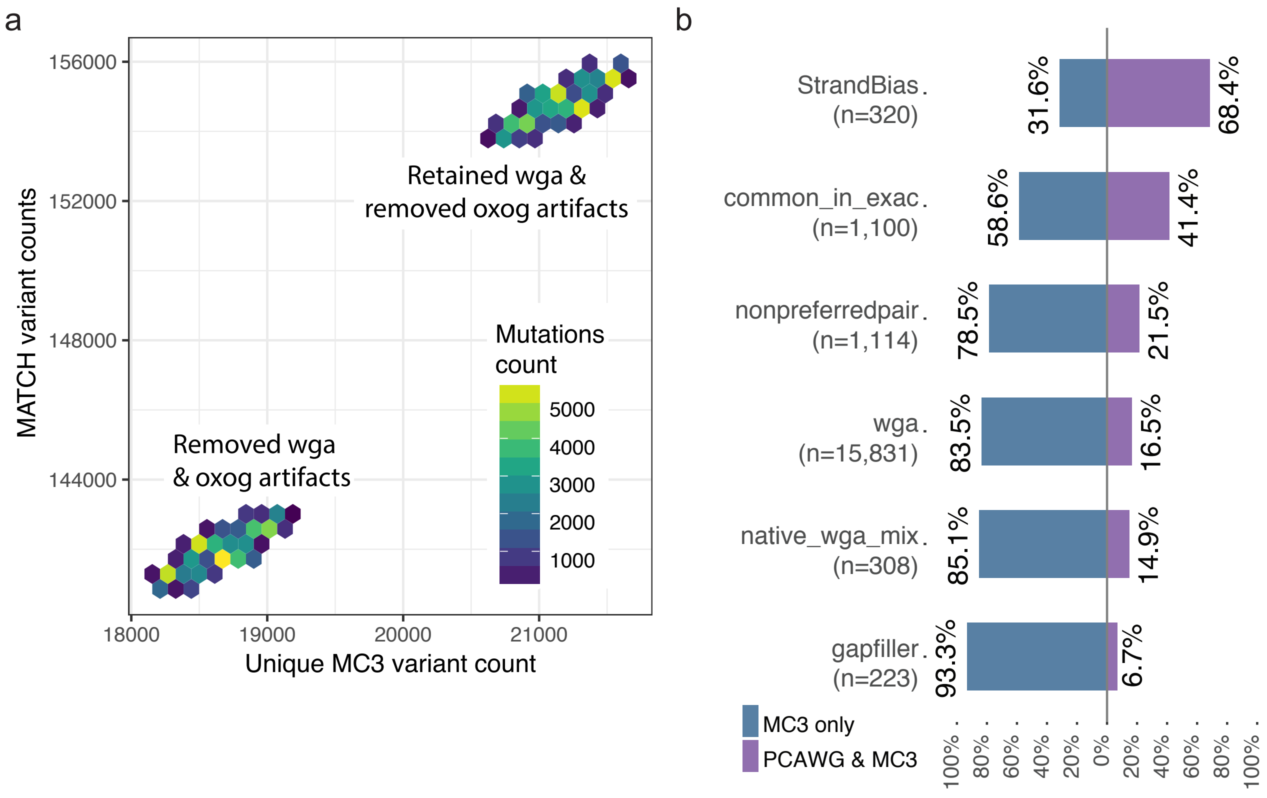

Supplementary Figure 3: Assessing MC3 filter flags. a) Displays results from an exhaustive search for an optimal set of MC3 filters. We performed an exhaustive search of MC3 filter combinations to identify an optimal set of MC3 filters to apply to our overlap criteria. This effort displayed using a resulted to two clusters of mutations with largely the same counts of MC3 unique calls (x-axis) and matched variant counts (y-axis). This result led to the decision to keep only remove OxoG artifacts from the overlap criteria. b) MC3 mutations with filter flags also observed in PCAWG data. A Likert[1] plot displays the proportion of flagged mutations provided unique to MC3 (blue) and the fraction of mutations present in both MC3 and PCAWG. Total mutations counts are labels below the name of each MC3 filter flag[2].

Supplementary Figure 4

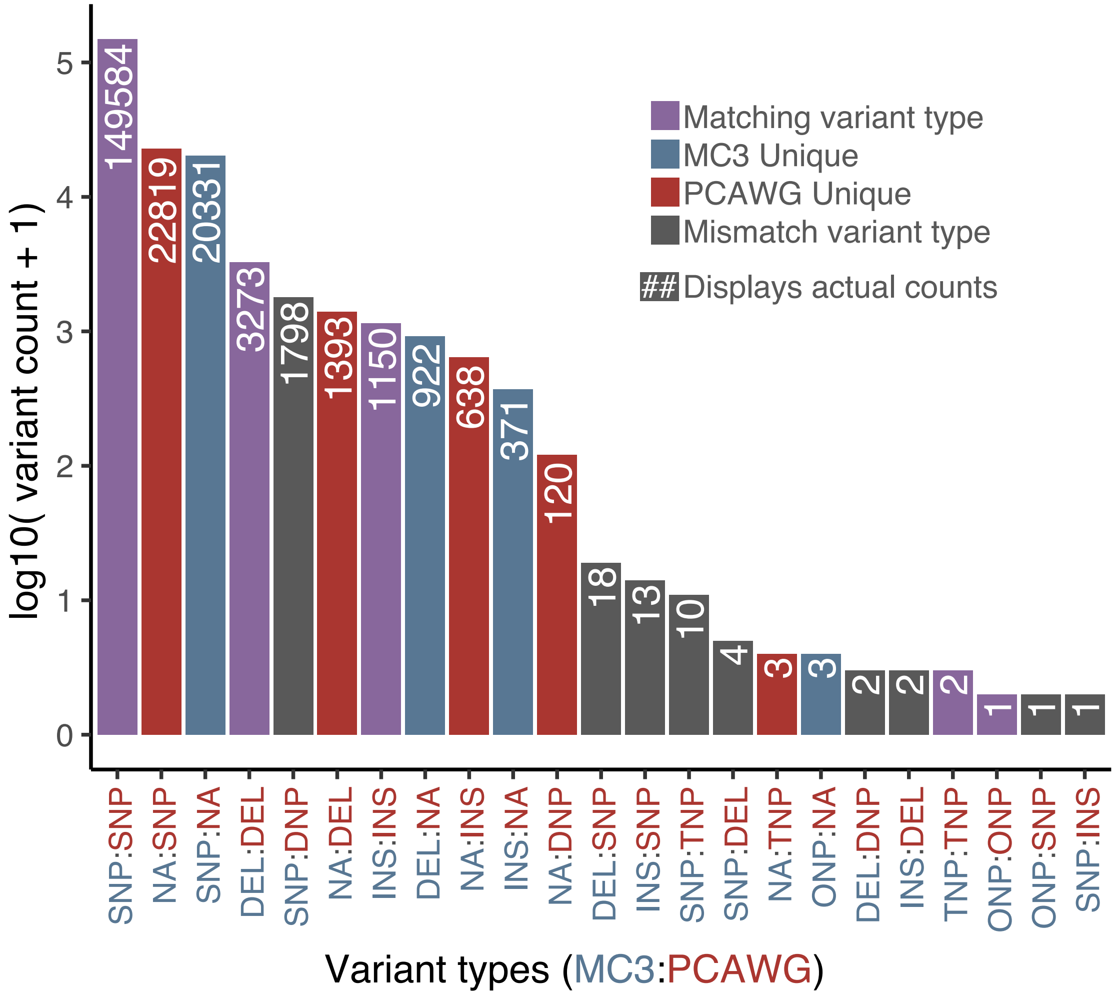

Supplementary Figure 4: Variant type breakdown. A bar chart shows the breakdown of unique, matched and mismatched variant types in the overlapping dataset. Bars that are purple represent variants with matching variant types in both samples as labeled in mutations annotation formatted files (.maf). Blue bars indicate variants unique to the MC3 data set. Red bars indicate the number of variants unique to the PCAWG data set. Grey bars indicate matched variants with different variant type annotations (x-axis). Actual counts are shown at the top of each bar in white. The y-axis is log_{10} scaled.

Supplementary Figure 5

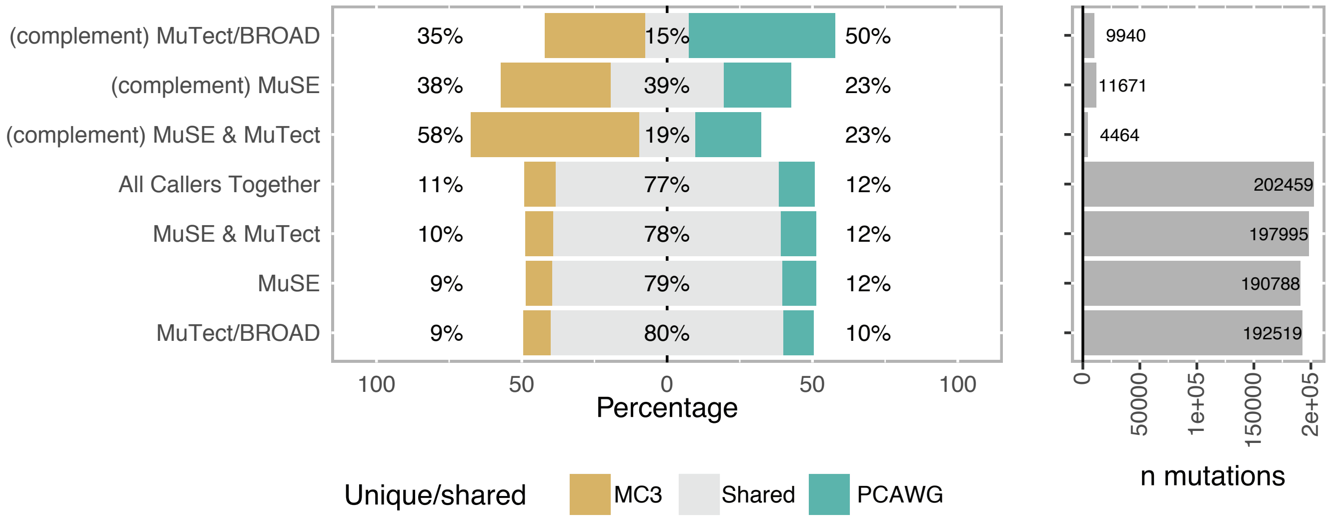

Supplementary Figure 5: Results of extracting subsets of MuSE and MuTect calls and their complements from the full set of mutation calls. Segmented barchart (left) shows that MuSE-only, MuTect-only, and their combined subsets have approximately 80% concordance. Barchart at right shows these 3 subsets each account for most of the calls of the full data set. Conversely, the complementary data-specific call sets (top 3 elements of the segmented chart) exhibit large variations, with concordance as low as 15%, and represent only small fractions of the total number of calls. Overall, differences in software pipelines between WES and WGS do not appear to be a significant confounding factor.

Supplementary Figure 6

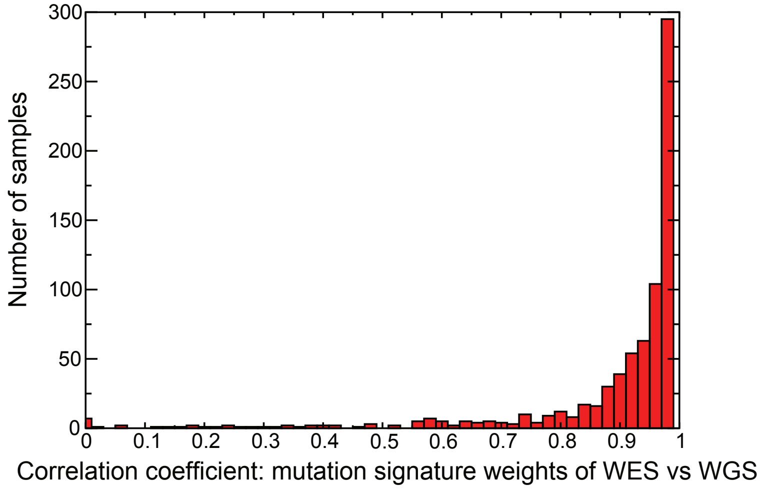

Supplementary Figure 6: Assessment of impact on mutation signature analysis. We used SignatureAnalyzer to predict mutation signatures for WES and WGS data and sought to assess how differences in mutation calls for these data would manifest in higher-level mutational signature analysis. Assessment is shown as a histogram of the Pearson correlation coefficient between the vectors of predicted signature weights from SignatureAnalyzer for matched WGS and WES samples for each of 739 cases. The majority, 555 (75%), show very strong correlation (coefficient at least 90%), with the average correlation for the cohort being 89.9%. These samples collectively show a cohort P-value $< 2 \times 10^{-6}$ (Fisher's transformation).

Supplementary Figure 7

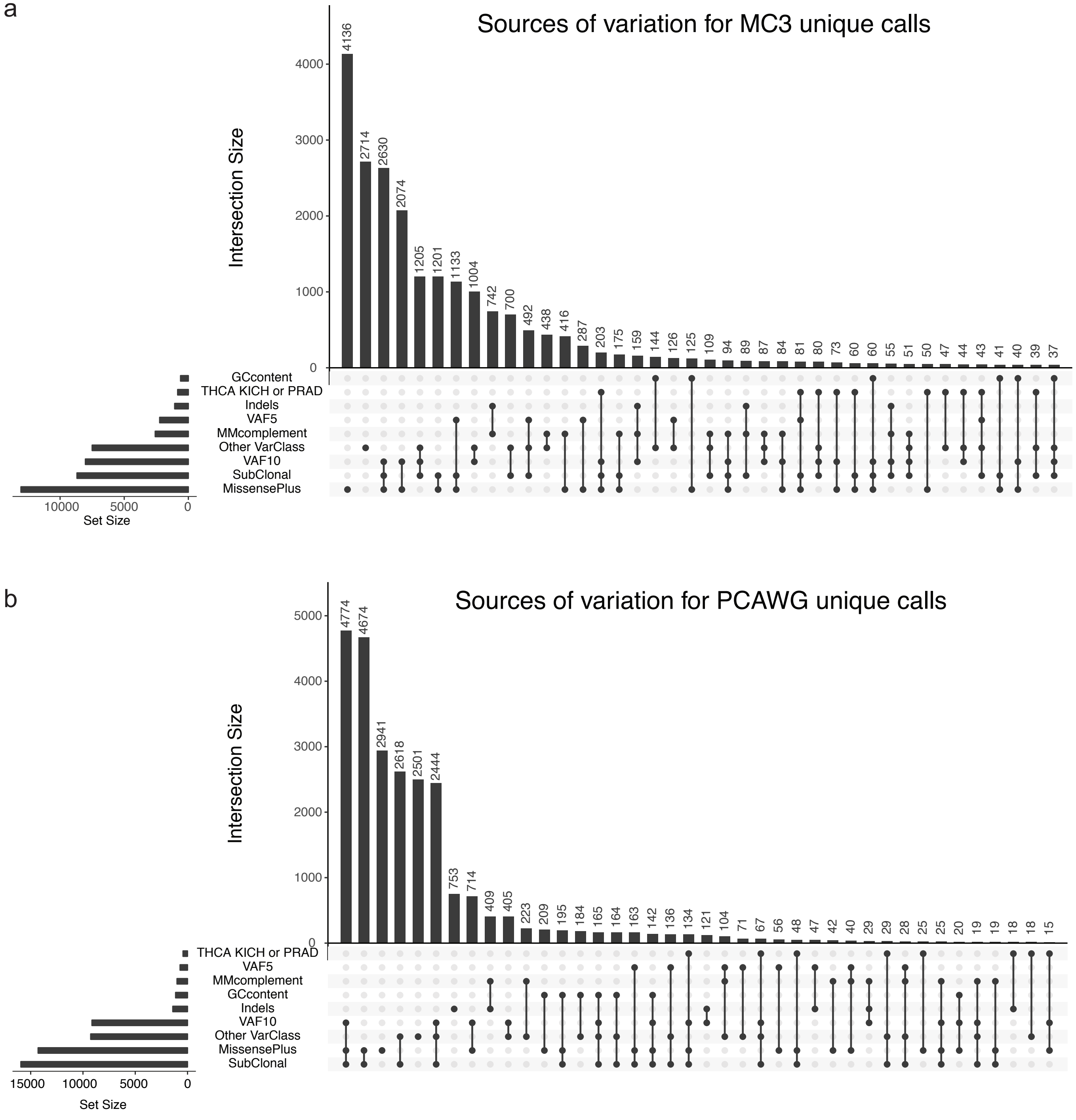

Supplementary Figure 7: Sources of variation in private WES calls, and private WGS calls. Two 'UpSetR'[3] plots were generated to delineate the origin of variation between unique calls in MC3(a) and PCAWG(b). 'UpSetR' diagrams provide a unique way to display overlapping sets. Here three variant classifiers sets are used: MissensePlus, Indels, and Other VarClass. An additional six variation source sets are used: GCcontent (GC fraction in 100bp window: 0.7 < GC-content < 0.3) , THCA KICH PRAD (poor performing cancer types), VAF5 (0% < VAF < 5%), VAF10 (5% <= VAF < 10%), SubClonal, MMcomplement (calls originating from callers other than MuSE or MuTect).

Supplementary Figure 8

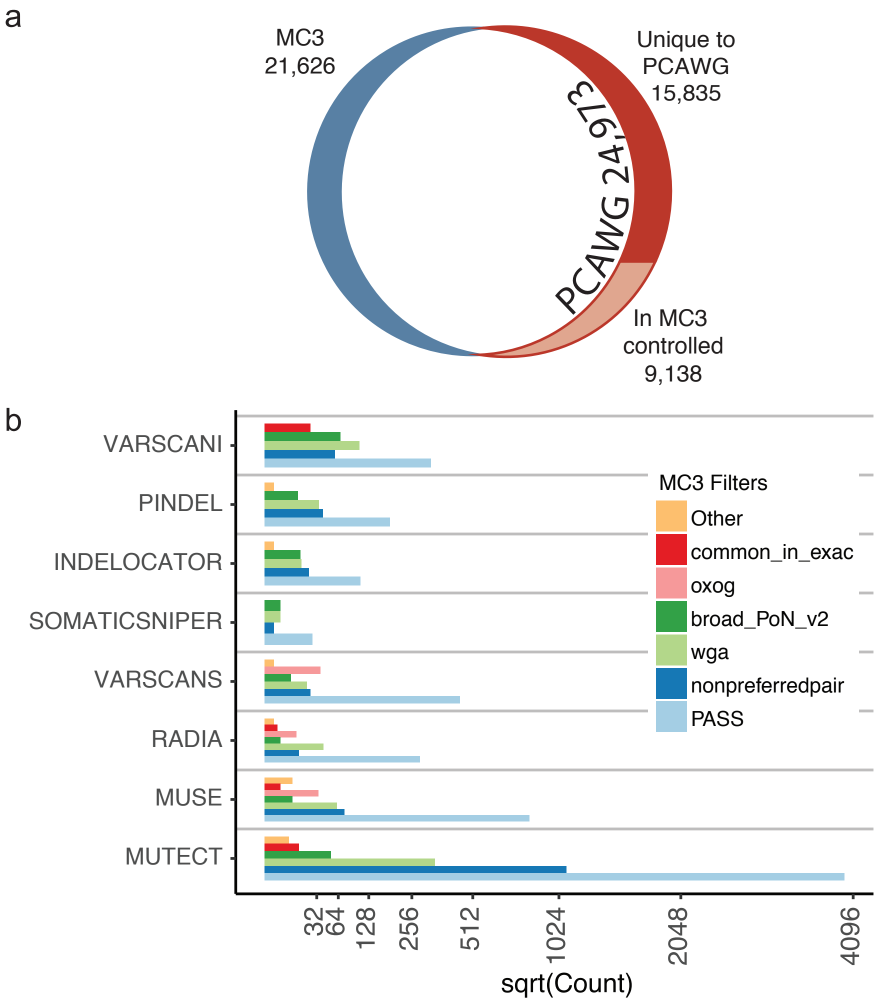

Supplementary Figure 8: Fraction and breakdown of unique PCAWG mutations also found in MC3 controlled dataset. a) The relative complements of the MC3/PCAWG Venn diagrams is shown. The right portion of the Venn diagram splits into two sub components in order to illustrate the number of PCAWG variants found in the MC3 controlled dataset. b) The set of 9138 unique PCAWG mutations also detected in the MC3 controlled dataset was further separated by MC3 variant caller and MC3 filter flags[2].

Supplementary Figure 9

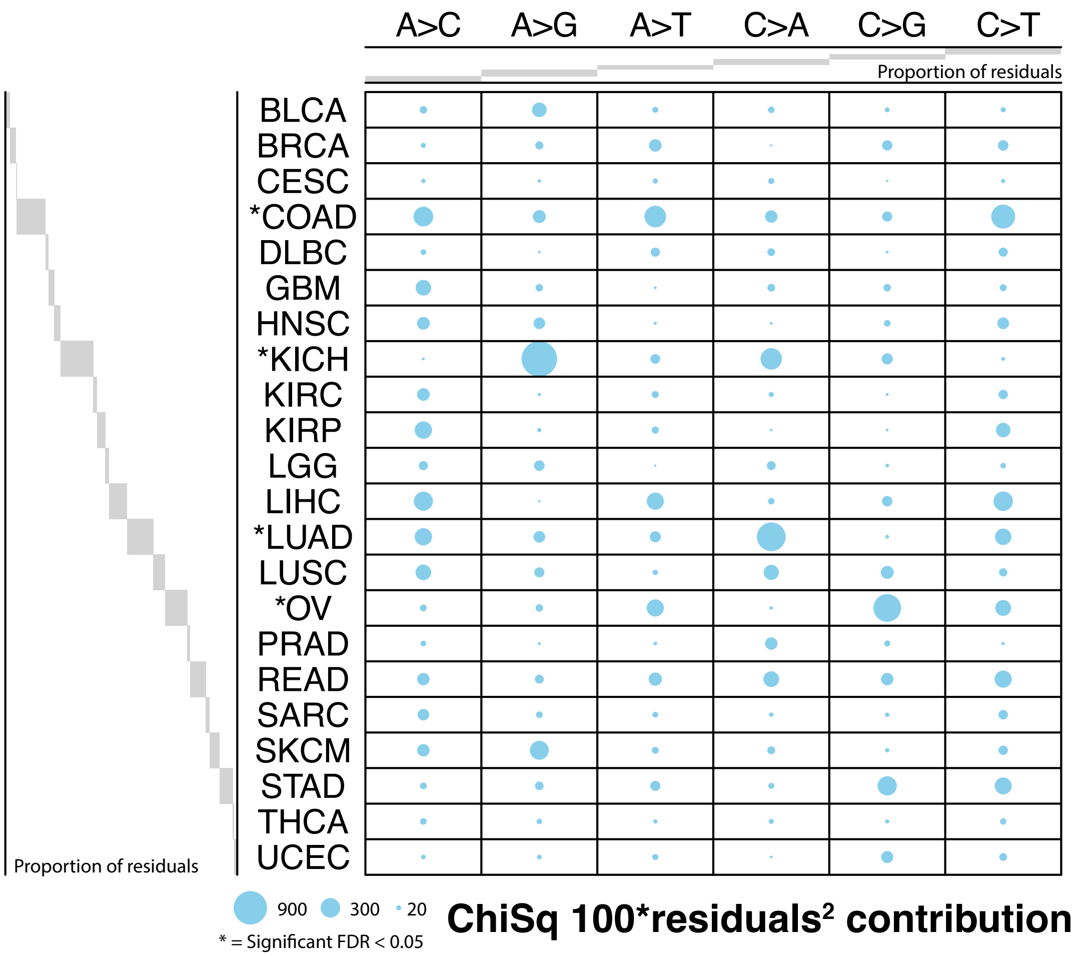

Supplementary Figure 9: Mutation spectrum analysis highlights differences between WGS and WES. Pearson's chi-squared residuals were calculated to compare the mutations spectrum across cancers from captured exomes versus whole genome sequencing of the same region. The analysis was performed by cancer type and the large dot indicates which transition or transversion contributed most to the spectrum difference. The size of the dot is 100-times the Pearson residual for a single transition or transversion.

Supplementary References

1. Bryer, J. & Speerschneider, K. likert: Functions to analyze and visualize likert type items. *R Packag. version* **1**, (2013).
2. Ellrott, K. *et al.* Scalable Open Science Approach for Mutation Calling of Tumor Exomes Using Multiple Genomic Pipelines. *Cell Syst.* **6**, 271-281.e7 (2018).
3. Conway, J. R., Lex, A. & Gehlenborg, N. UpSetR: An R package for the visualization of intersecting sets and their properties. *Bioinformatics* **33**, 2938–2940 (2017).
