# Supplementary material for: Retrospective evaluation of whole exome and genome mutation calls in 746 cancer samples
Source: Nat Commun. 2020 Sep 21;11:4748. doi: 10.1038/s41467-020-18151-y (PMC7505971; doi:10.1038/s41467-020-18151-y)
Supplement: Supplementary file 2 — Description of Additional Supplementary Files [file 41467_2020_18151_MOESM2_ESM.pdf]

Description of Additional Supplementary Files

File Name: Supplementary Data 1
Description: Mapping MC3 and PCAWG sample identifiers

File Name: Supplementary Data 2
Description: Filter criteria for variant filter flags

File Name: Supplementary Data 3
Description: TCGA subtype selection preferences to WGS sequencing

File Name: Supplementary Data 4
Description: Misannotated variants between WES and WGS

File Name: Supplementary Data 5
Description: Mutation spectrum comparison by cancer

File Name: Supplementary Data 6
Description: WGS specific cancer mutations
